# Is Western Diet-Induced Nonalcoholic Steatohepatitis *in Ldlr^-/-^* Mice Reversible?

**DOI:** 10.1371/journal.pone.0146942

**Published:** 2016-01-13

**Authors:** Kelli A. Lytle, Donald B. Jump

**Affiliations:** 1 Nutrition Program, School of Biological and Population Health Sciences, Oregon State University, Corvallis, Oregon, United States of America; 2 Linus Pauling Institute, Oregon State University, Corvallis, Oregon, United States of America; INRA, FRANCE

## Abstract

**Background:**

Nonalcoholic fatty liver disease (**NAFLD**) is a major public health burden in western societies. The progressive form of NAFLD, nonalcoholic steatohepatitis (**NASH**), is characterized by hepatosteatosis, inflammation, oxidative stress, and hepatic damage that can progress to fibrosis and cirrhosis; risk factors for hepatocellular carcinoma. Given the scope of NASH, validating treatment protocols (i.e., low fat diets and weight loss) is imperative.

**Methods:**

We evaluated the efficacy of two diets, a non-purified chow (**NP**) and purified (low-fat low-cholesterol, **LFLC**) diet to reverse western diet (**WD**)-induced NASH and fibrosis in *Ldlr*^*-/-*^ mice.

**Results:**

Mice fed WD for 22–24 weeks developed robust hepatosteatosis with mild fibrosis, while mice maintained on the WD an additional 7–8 weeks developed NASH with moderate fibrosis. Returning WD-fed mice to the NP or LFLC diets significantly reduced body weight and plasma markers of metabolic syndrome (dyslipidemia, hyperglycemia) and hepatic gene expression markers of inflammation (*Mcp1*), oxidative stress (*Nox2*), fibrosis (*Col1A*, *LoxL2*, *Timp1*) and collagen crosslinking (hydroxyproline). Time course analyses established that plasma triglycerides and hepatic *Col1A1* mRNA were rapidly reduced following the switch from the WD to the LFLC diet. However, hepatic triglyceride content and fibrosis did not return to normal levels 8 weeks after the change to the LFLC diet. Time course studies further revealed a strong association (r^2^ ≥ 0.52) between plasma markers of inflammation (TLR2 activators) and hepatic fibrosis markers (*Col1A*, *Timp1*, *LoxL2*). Inflammation and fibrosis markers were inversely associated (r^2^ ≥ 0.32) with diet-induced changes in hepatic ω3 and ω6 polyunsaturated fatty acids (**PUFA**) content.

**Conclusion:**

These studies establish a temporal link between plasma markers of inflammation and hepatic PUFA and fibrosis. Low-fat low-cholesterol diets promote reversal of many, but not all, features associated with WD-induced NASH and fibrosis in *Ldlr*^*-/-*^ mice.

## Introduction

Nonalcoholic fatty liver disease **(NAFLD)** is the most common cause of chronic fatty liver disease in the United States affecting 10–35% of adults and an increasing number of children [1–3]. The incidence of NAFLD is especially high (≥60%) in the obese and type 2 diabetic populations. NAFLD is primarily characterized by excess deposition of neutral lipid (hepatosteatosis) in the liver in which >5% of the liver is stored neutral lipid (triglycerides & cholesterol esters). Hepatosteatosis can progress to nonalcoholic steatohepatitis **(NASH).** NASH is characterized by hepatic inflammation, hepatocyte damage/death and oxidative stress. Excessive damage to the liver promotes fibrosis, i.e., deposition of extracellular matrix (**ECM**), consisting of collagens, elastin and other proteins. NASH-associated fibrosis is a risk factor for cirrhosis and primary hepatocellular carcinoma. Complications arising from NASH are projected to be the leading cause of liver transplants by 2020 [4].

Currently there are no FDA approved therapies for NAFLD and generally clinicians simply treat underlying comorbidities associated with metabolic syndrome **(MetS),** i.e., obesity and fasting hyperglycemia and dyslipidemia. Given that NAFLD/NASH is characterized by hepatic lipid deposition and often accompanied by central obesity, it is a logical recommendation for clinicians to encourage their patients to alter dietary intake and lose excess body weight. However, the efficacy of these recommendations on advanced NASH is not well characterized.

Moreover, a complication of advanced NASH is the development of fibrosis resulting from significant liver injury. While fibrosis was once considered irreversible, more recent studies in rodents and humans indicate that hepatic fibrosis is reversible [5–12]. The general findings from these studies suggest that hepatic fibrosis resolves once the stimulus for liver injury is removed. These studies, however, examined the reversibility of fibrosis in the absence of the chronic metabolic phenotype that characterizes NASH in the obese-MetS patient. As such, the applicability of these findings to obese-MetS patient with NASH is unclear.

To address this issue, we developed a mouse model using *Ldlr*^*-/-*^ mice fed the western diet **(WD)** [13–15]. The WD is moderately high (~43% total calories) in saturated, monounsaturated and trans-fat, sucrose (30% total calories), and cholesterol (0.15 gm%). Long-term WD feeding induces a severe NASH phenotype in the context of MetS in *Ldlr*^*-/-*^ mice; in which *Ldlr*^*-/-*^ mice become obese and display metabolic markers of MetS, such as fasting hyperglycemia and dyslipidemia. Plasma from these mice has revealed evidence of endotoxinemia and hepatic damage (ALT and AST) and livers from these mice exhibit histological and biochemical evidence of being fatty, inflamed and fibrotic. The NASH phenotype in these mice develops in conjunction with the onset of MetS, obesity, hyperglycemia, dyslipidemia and insulin resistance. As such this mouse model is representative of human diet-induced NASH and recapitulates the phenotype of NASH in obese humans with MetS.

To test the hypothesis that weight loss and dietary change is efficacious in reversing NASH induced in the context of MetS, we determined the capacity of a non-purified chow (**NP**) and a purified low-fat low-cholesterol (**LFLC**) diet, typically used as a control diet in diet-induced obesity studies [16], to reverse WD-induced MetS and NASH in *Ldlr*^-/-^ mice. We used a time course approach to establish associations between changes in plasma and hepatic parameters during diet-induced remission of MetS and NASH. The outcome of this analysis establishes that switching WD-fed *Ldlr*^*-/-*^ mice to the NP or LFLC diet has major effects on many, but not all, features associated with MetS and NASH.

## Materials and Methods

### Animals and Diets

All procedures for the use and care of animals for laboratory research were approved by the Institutional Animal Care and Use Committee at Oregon State University. Male *Ldlr*^*-/-*^ mice [B6;129S7-*Ldlr*^*tm1Her*^/J, stock# 002207, purchased from Jackson Labs] were individually housed, maintained on a 12-hour light/dark cycle and were acclimatized to the animal facilities at OSU for 1-week before proceeding with experiments. At the termination of both studies, all mice were fasted overnight (1800h to 0800 h) prior to sacrifice by CO_2_ administration and exsanguination; blood and liver were collected as previously described [14].

#### Study 1: Effect of non-purified chow diet on remission of WD induced NASH

At 10 weeks of age mice were randomized to 3 treatment groups (4 mice per group): Group 1) fed *ad libitum* Purina Pico Lab Diet 5053 for 29 wks [(non-purified diet, [**NP**], **S1 Table**]; Group 2) fed *ad libitum* Western Diet (**WD**, Research diets D12079B) for 29 wks [**WD-29**]; Group 3) mice were fed *ad libitum* WD for 22 wks and then switched to *ad libitum* NP diet for 7 wks **(WD to NP).** All mice were sacrificed after 29 wks on the NP or WD diets.

#### Study 2: Effect of purified low-fat low-cholesterol diet on remission of WD induced NASH

Study 2 consisted of 6 groups (5–6 mice per group). At 9 wks of age mice were fed one of two diets: the WD or the low-fat low-cholesterol (**LFLC**) diet (Research Diets 12450B). Sucrose content in the LFLC diet nearly matched the sucrose content in the WD (**S1 Table**). Mice were maintained on their respective diets for 24 wks. This study assessed the effect of reduced dietary lipid (fat and cholesterol) on NASH remission. A WD-fed group was sacrificed after 24 wks on the diet to establish baseline hepatic status (**WD-24**). At 24 wks additional WD-fed groups were switched to the LFLC diet (**WD-24 to LFLC**) for 1, 2 or 8 wks; (i.e., **WD-24 to LFLC 1 wk**; **WD-24 to LFLC 2 wks**; **WD-24 to LFLC 8 wks**, respectively). LFLC-fed mice (**LFLC-32**) and WD-fed mice (**WD-32**) were maintained on their respective diets for 32 wks.

A power calculation (http://www.dssresearch.com/toolkit/spcalc/power_a1.asp) was carried out with the following parameters: difference between the test (test value = 8) and control (control value = 4) i.e., mean difference is 2-fold; standard deviation 20% of the mean; 95% confidence, the statistical power for 4 and 6 animals (sample size) was 99.1% and 99.9%, respectively.

### RNA Extraction and qRT-PCR

RNA was extracted from livers using Trizol (Life Technologies) as described [16]. RNA was quantified via spectrophotometry using a nanodrop-1000. qRT-PCR was performed using the 7900HT fast machine from Applied Biosystems as previously described [16].

### Hepatic Lipid Composition

Hepatic lipids were extracted as previously described [17]. Total lipid extract was subject to saponification and methylation; and fatty acid methyl esters were separated and quantified by gas chromatography [17]. GC standards were purchased from Nu-Chek Prep Inc. Hepatic protein content was measured using Quick Start Bradford Reagent (Bio-Rad) and bovine serum albumin (Sigma-Aldrich) as a standard.

### Plasma and Hepatic Measures

Plasma triglycerides, total and free cholesterol and glucose were measured using kits obtained from Wako. Plasma aspartate amino transferase **(AST)** and alanine amino transferase **(ALT)** were measured using kits from Thermo Fischer Scientific. Plasma endotoxin was measured using a kinetic chromogenic kit obtained from Lonza. Plasma Toll Like receptor **(TLR)**2 and TLR4 agonist activity was measured using Hek-Blue cell systems from Invivogen. Plasma leptin and adiponectin were measured with ELISA kits from R&D Systems. Hepatic hydroxyproline was measured using a colorimetric assay obtained from Sigma-Aldrich according to manufactures protocol and normalized to hepatic protein content as measured by the Bradford Assay.

### Liver Histology

Liver (~100 mg) was fixed in buffered-formalin, paraffin embedded, sliced, and stained with hematoxylin-eosin, trichrome or Sirius red (Nationwide Histology, Veradale, WA). Each slide contained 4–6 slices/liver. Steatosis and fibrosis was seen consistently on all liver sections from the same animal. Photomicrographs of liver sections shown in the figures are representative of all livers within each group.

### Heat maps, volcano plots and statistical analysis

Heat maps were prepared using data on body weight, plasma (glucose, lipids, ALT, AST, leptin, adiponectin, TLR2 and TLR4 agonist), and hepatic (triglycerides, cholesterol, fatty acid profiles, gene expression, hydroxyproline). The data were analyzed using the statistical package in MetaboAnalyst 3.0 [http://www.metaboanalyst.ca/MetaboAnalyst/] [18]. The analysis generated heat maps, volcano plots, correlation analyses and ANOVA with Tukey’s HSD Post-hoc test. We also used a separate online statistical package [http://vassarstats.net/] for one-way ANOVA with Tukey’s HSD Post-hoc test of specific features to detect significant differences between groups when more than two groups were included in the analysis. Student’s t-test was used when only two groups were being compared and non-parametric tests were used when unequal variance as determined by f-test was detected between two groups. A p-value ≤0.05 was considered statistically different. All values are reported as mean ± SD.

## Results

### The NP diet partially reverses WD-induced NASH and fibrosis in *Ldlr*^*-/-*^ mice

**Fig 1:**
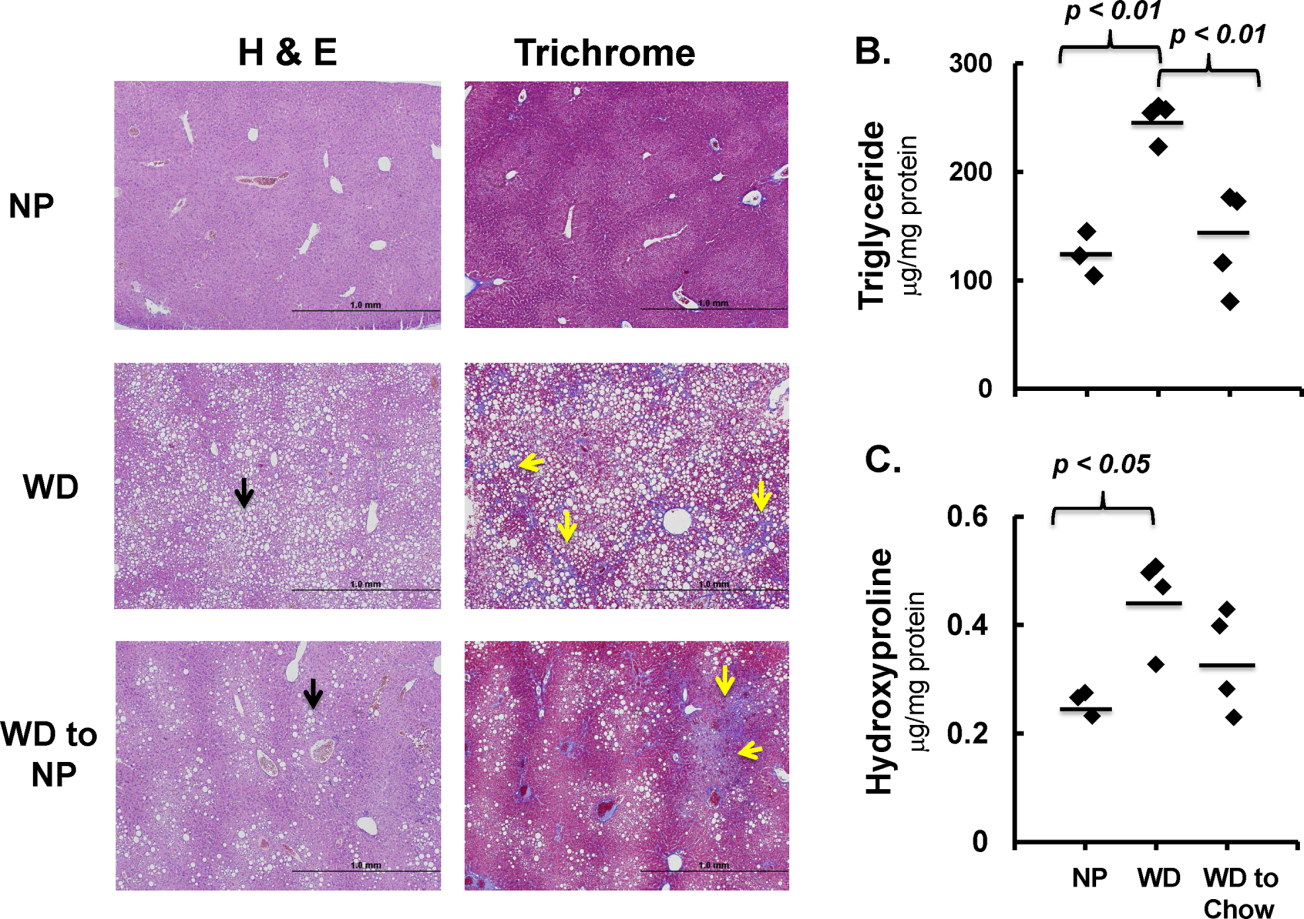
Effects of non-purified lab (NP) diet on reversal of WD-induced NASH. Mice were fed the NP (Lab Chow) or WD diets as described in Materials and Methods. **[A]:** The left and right panels represent hematoxylin-eosin (H&E) and trichrome (TC) staining of liver sections, respectively. Magnification for the H & E and TC panels was 4X, respectively. Lipid droplets appear as white circles (black arrows), while branching fibrosis (yellow arrows) appears as blue strands in the trichrome stained liver sections. The slides are representative of all livers in each group. **[B]:** Hepatic triglyceride was quantified as described in Methods and Materials. Results are represented as Triglyceride, μg/mg protein. **[C]:** Hepatic hydroxyproline content was quantified as described in Methods and Materials. Results are represented as Hydroxyproline μg/mg protein. Statistical analysis used ANOVA plus Tukey’s HSD to establish statistical significance.

Feeding *Ldlr*^*-/-*^ mice the WD for 29 wks induced a MetS and NASH phenotype characterized by obesity, dyslipidemia, hepatosteatosis and fibrosis (**Fig 1 and S2 Table; S1 and S2 Figs**). Hepatic triglycerides (a marker of steatosis) and hydroxyproline (a marker of fibrosis and collagen cross-linking) were elevated in WD-fed mice ~2.5 and ~2-fold, respectively. Increased hepatic hydroxyproline levels correlated with histological evidence of branching fibrosis (Trichrome: blue stained collagen, yellow arrows, **Fig 1**).

After 22 wks on the WD, mice were switched to the NP diet for 7 wks. This change in diet lowered body weight; hepatic triglycerides to levels seen in mice fed the NP for 29 wks. While hepatic hydroxyproline levels were reduced in this group, this change in hydroxyproline was not significant. Trichrome staining also detected evidence of fibrosis in livers of mice switched from the WD to NP diet. While switching the diet from the WD to NP succeeded in significantly reducing body weight and hepatosteatosis, it failed to fully reverse WD-induced hepatic fibrosis.

**Fig 2:**
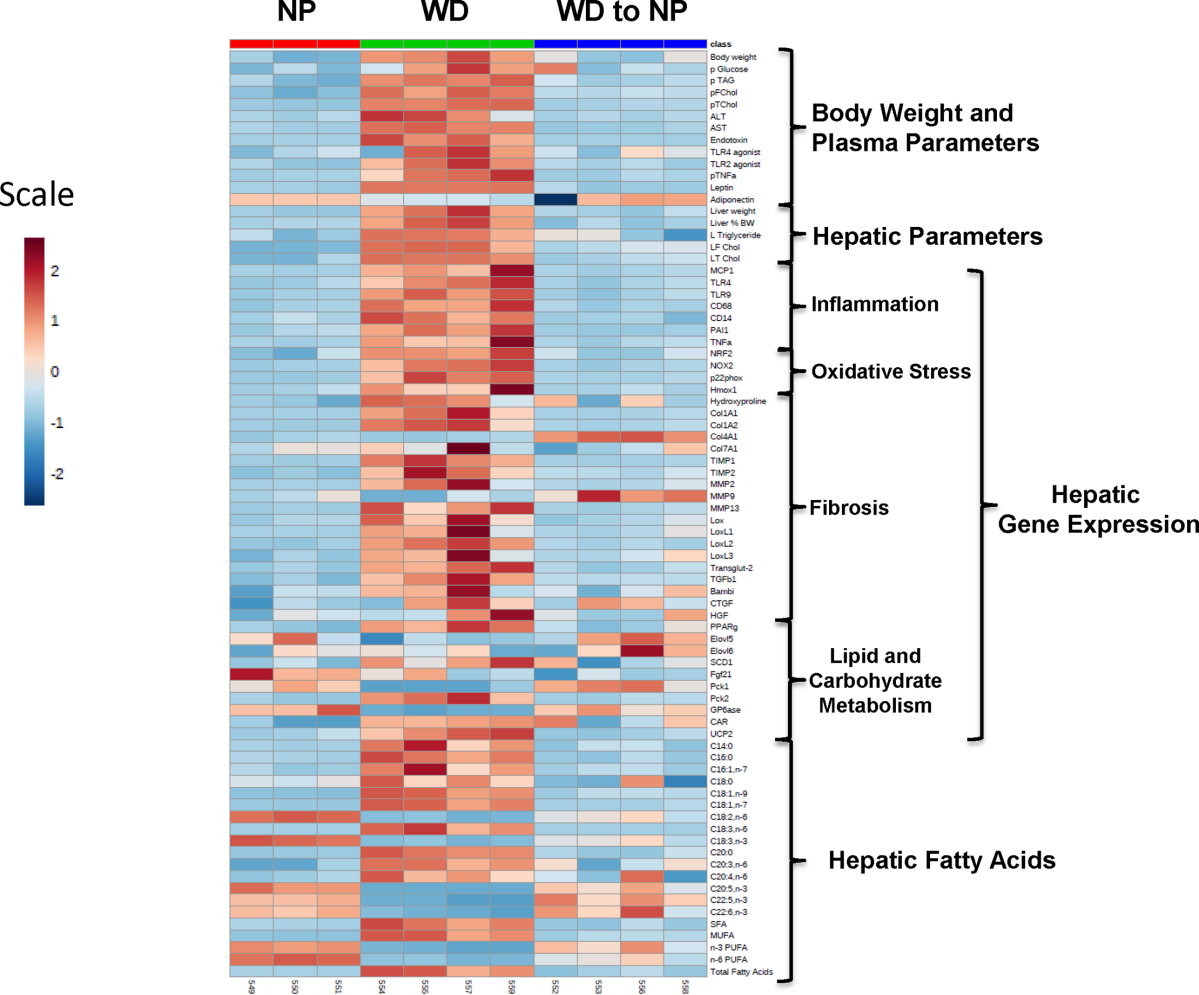
Heat map of changes in whole body, plasma and hepatic features associated with the NP, WD-29 and WD to NP groups. The heat map was created using the statistical package in MetaboAnalyst 3.0 (http://www.metaboanalyst.ca/MetaboAnalyst/) as described in Materials and Methods. **[A]**: The heat map is a visualization of the changes in abundance/level of features in rows (e.g., plasma glucose) for each animal (columns). The number at the bottom of the heat map is the animal identification number. The color ranges from deep orange (high abundance or level) to deep blue (low abundance or level); white is no change.

We next determined if changes in histologic features paralleled changes in other physiological NASH markers. The heat map (**Fig 2**) includes data on body weight, plasma and hepatic parameters, as well as hepatic fatty acids. Data used for the heat map is displayed in **S2 Table; S1 and S2 Figs**. Most parameters increased in mice fed the WD, when compared to mice fed the NP diet; this included body weight, blood glucose, and plasma levels of lipids, TLR2 and TLR4 activators, markers of hepatic damage (ALT & AST), TNFα and leptin. Hepatic lipids (triglyceride & cholesterol) and NASH gene expression markers associated with inflammation; and hepatic saturated and monounsaturated fatty acid content was also increased. Factors decreasing in response to the WD feeding included plasma adiponectin, hepatic enzymes involved in hepatic ECM remodeling (*Col4A1*, *Mmp9*), fatty acid elongation (*Elovl5 and Elovl6*), carbohydrate metabolism (*Pck1*, *G6Pase*) and hepatic levels of ω3 and ω6 PUFA.

In mice fed the WD for 22 weeks and switched to the NP for 7 weeks, nearly complete reversal of all parameters was observed. Taken together, this data suggests that a diet low in fat, simple sugar and cholesterol can reverse many of the whole body, plasma and hepatic features associated with WD-induced NASH. However, some fibrosis persists in this group (**Fig 1**).

### Purified low-fat low-cholesterol (LFLC) diet partially reverses WD-induced NASH

The NP diet used in Study 1 is a rodent maintenance diet and is not characteristic of human diets. Study 2 assessed a purified low-fat low-cholesterol (**LFLC**) diet typically used as a control diet in diet-induced obesity studies with wild type C57BL/6J mice [16, 19]. This study was also designed to determine the time course across which the LFLC diet feeding resolved physiological markers of NASH.

**Fig 3:**
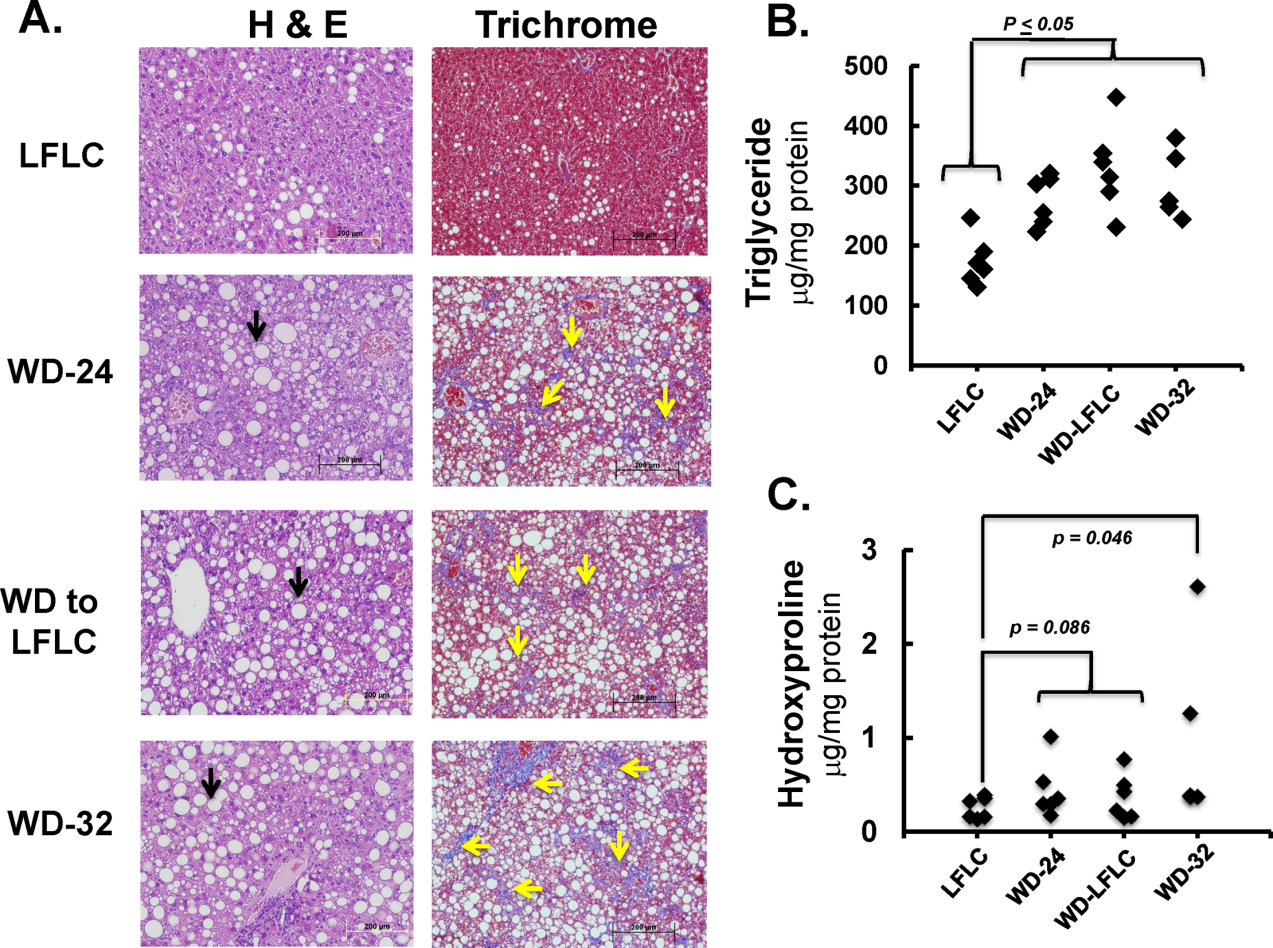
Can a LFLC diet reverse WD-induced NASH? Mice were fed the LFLC or WD diets as described in Materials and Methods. **[A]**: The left and right panels represent hematoxylin-eosin (H & E) and trichrome (TC) staining of liver sections, respectively. Magnification for the H & E and TC panels was 16X and 10X, respectively. Lipid droplets appear as white circles (black arrows), while fibrosis (branching) appears as blue strands (yellow arrows) in the trichrome stained liver sections. The slides are representative of all livers in each group. **[B]**: Hepatic hydroxyproline content was quantified as described in Materials and Methods. Results are represented Hydroxyproline μg/mg protein

*Ldlr*^*-/-*^ mice were fed the WD for 24 wks (WD-24) and extended feeding for 32 wks (WD-32). As with Study 1, these mice gained weight and exhibited multiple plasma and hepatic features associated with MetS and NASH (**Fig 3, S3 Table**). The plasma markers of this phenotype included: hyperglycemia, dyslipidemia and elevated plasma levels of ALT, leptin, TNFα and TLR2 and TLR4 activators. Livers of these mice were engorged with lipid and displayed evidence of mild fibrosis, as reflected by increased blue staining in trichrome stained livers and elevated hydroxyproline levels (**Fig 3**).

Switching mice from the WD (WD-24) to the LFLC diet for 8 wks (WD-LFLC-8 wks) corrected many but not all, of the effects of 24 wks of WD feeding. Specifically, this intervention reduced body weight, blood glucose, and plasma levels of lipids, ALT, leptin and TLR2 and TLR4 agonists to levels seen in control mice maintained on the LFLC diet for 32 wks. However, livers from WD-LFLC-8 mice weighted more and had greater levels of triglyceride and cholesterol than control mice fed the LFLC diet for the 32 wks. Hepatic fibrosis, as measured by hydroxyproline, was not different between WD-fed mice for 24 weeks and WD-LFLC-8 wk and was greater than mice fed the LFLC diet for the 32 weeks. Consistent with this data, histological analysis (trichrome staining; **Fig 3)** indicate that branching fibrosis (yellow arrows in **Fig 3**) is still present in livers of WD-LFLC-8 wk mice. As such, the LFLC diet does not promote full remission of NASH or fibrosis.

A phenotypic comparison of *Ldlr*^*-/-*^ mice fed the two control diets (NP and LFLC) for 29 and 32 wks, respectively revealed that mice fed the LFLC diet for 32 wks weighed more (23%) and had higher plasma parameters associated with MetS, hepatic damage and systemic inflammation than NP fed mice (**S4 Table**). Livers of these mice did not differ in terms of overall weight, liver weight as a % of body weight, or triglyceride content, but had increased (72%) hepatic cholesterol. Gene expression markers of inflammation (*Mcp1*) and fibrosis (*Col1A1*, *Timp1*) in livers of the LFLC-fed mice are significantly elevated when compare to mice fed the NP diet.

### Physiological changes occurring with WD-induced NASH

**Fig 4:**
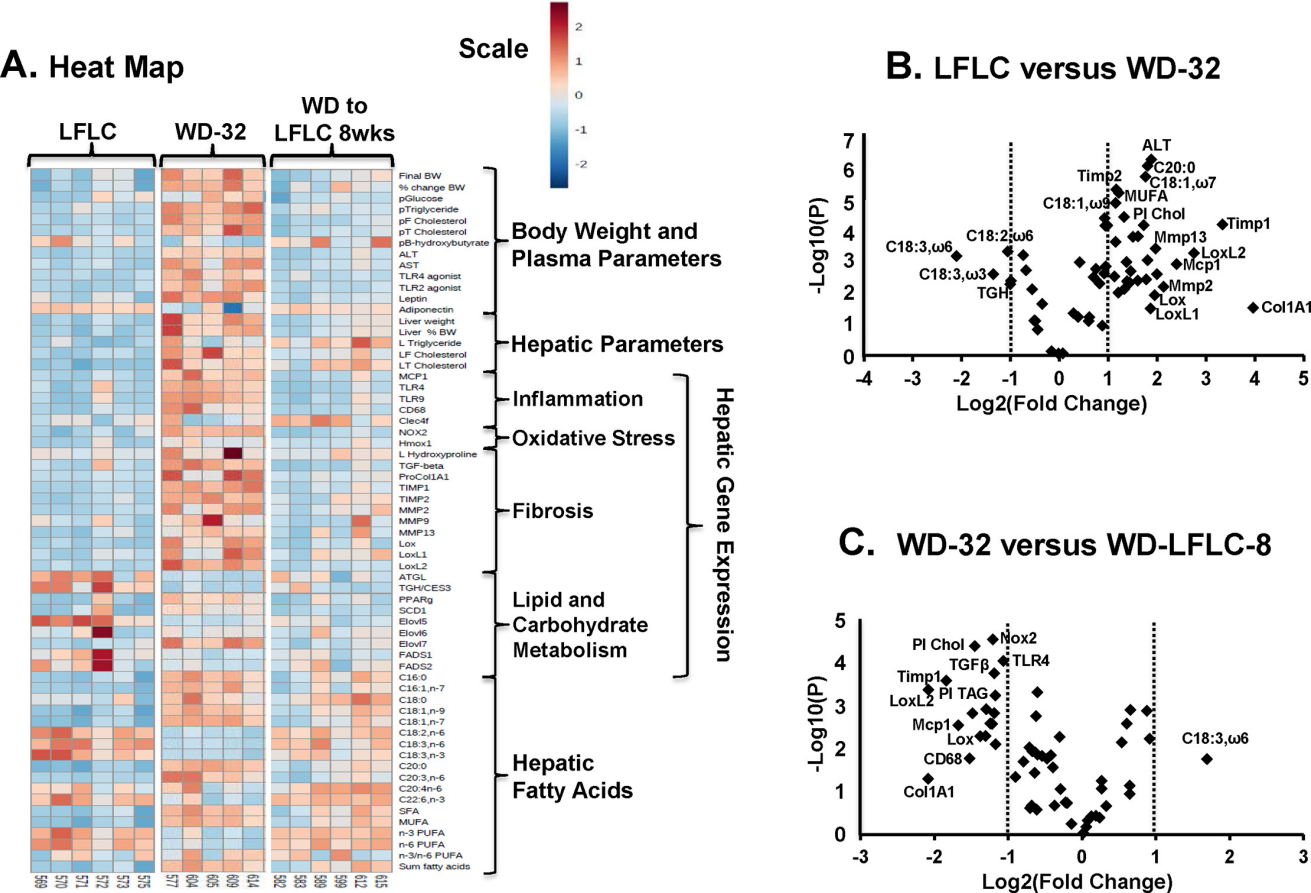
Heat maps and volcano plots of the LFLC, WD-32 and WD to LFLC groups. Bioinformatic analysis of data used the statistical package in MetaboAnalyst 3.0 (http://www.metaboanalyst.ca/MetaboAnalyst/) as described in Materials and Methods. **[A]:** The heat map is a visualization of the changes in abundance/level of features in rows (e.g., plasma glucose) for each animal (columns). The number at the bottom of the heat map is the animal identification number. The color ranges from deep orange (high abundance or level) to deep blue (low abundance or level); white is no change. **[B and C]:** All data in the heat map was used to construct volcano plots using the statistics package in MetaboAnalyst 3.0. The volcano plot allows for the visualization of the distribution of p-values versus fold-change for all measured entities, which include body weight, plasma and hepatic parameters, hepatic transcripts and fatty acids.

The WD impacts a broad array of features associated with MetS and NASH (**Fig 4)**. Using a heat map and volcano plots, we compared the LFLC group, WD-32 and the WD-LFLC-8 wk groups (**Fig 4 and S3 Table).** The heat map illustrates that WD feeding was associated with alterations in body & liver weight, multiple plasma parameters associated with MetS, hepatic fatty acid content and numerous changes in hepatic gene expression associated with MetS and NASH. Hepatic gene expression markers of inflammation *(Mcp1*, *Cd68*, *Trl4)*, oxidative stress (*Nox2*, *Hmox1*), fibrosis (*Col1A1*, *Mmp2*, *Timp1 & 2*), as well as hepatic hydroxyproline and monounsaturated fatty acid content (16:1,ω7; 18:1,ω7; 18:1,ω9) were all greatly increased by WD diet feeding. As seen in Study 1, several features declined with NASH, including the expression of genes involved in triglyceride catabolism (*ATGL*, *TGH/Ces3*), fatty acid elongation (*Elovl5)* and hepatic ω3 and ω6 PUFA content. The volcano plot provides information on the magnitude of change (Log 2 Fold Change) and the p-values [-Log10(P)] associated with these changes. All of the plasma (ALT, cholesterol), hepatic inflammation (*Mcp1*), fatty acid (18:1,ω7, 20:0) and fibrosis markers (*Timp1*, *Mmp2*, *Mmp13*, *LoxL2*, *Col1A1*) markers were significantly induced by the WD (**Fig 4B**).

To determine the effects of 8 wks of LFLC diet on the remission of NASH, we compared the WD-32 and WD-LFLC-8 wks treatments groups (**Fig 4A & 4C**). The heat map and volcano plots show that most features that were induced by WD feeding were reversed by feeding the NASH mice the LFLC diet for 8 wks. In contrast to Study 1 where WD-fed mice were switched to the NP diet, the WD-fed mice switched to LFLC-8 wks group (Study 2) did not have a reduction in hepatic steatosis (triglyceride and cholesterol) (**Figs 3 & 4**) indicating that the two diets used to reverse WD-induced NASH did not yield entirely the same hepatic outcomes. The finding that neither control diet was capable of reversing WD induced hepatic fibrosis, suggests that reduction of hepatic fibrosis requires actions in addition to those induced by weight loss and the consumption of a low-fat low-cholesterol diet.

### Time course for diet effects on MetS and NASH features

**Fig 5:**
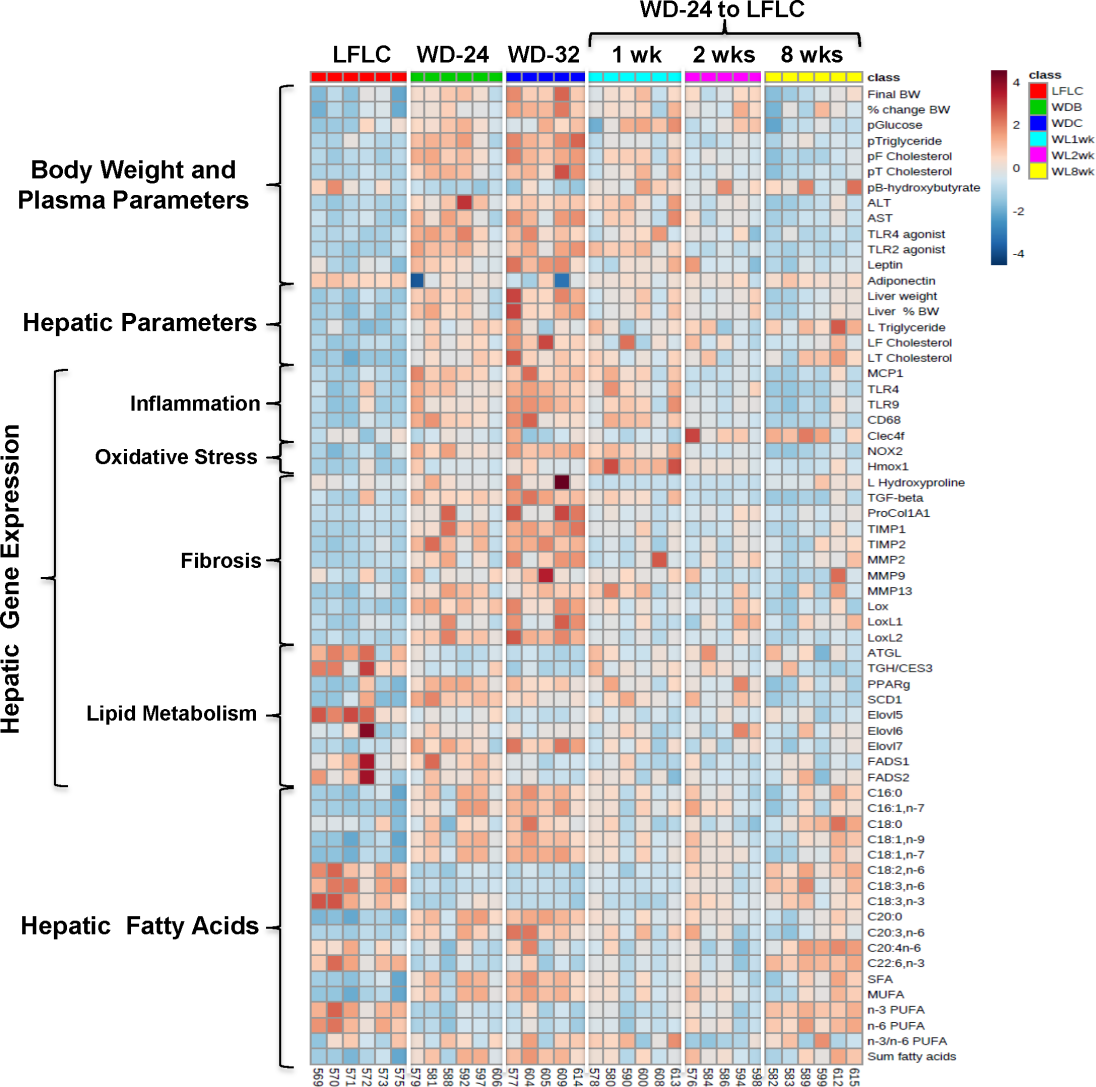
Heat map of the time course of diet effects on reversal of MetS and NASH. As explained in Fig 2, the heat map is a visualization of changes in entities (rows, e.g., plasma glucose) in mice (columns). This figure represents an analysis of the 6 groups in the time course study: LFLC, WD-24, WD-32, WD-24 to LFLC for 1, 2, or 8 weeks. The number of mice in each group is: 6, 5, 5, 6, 6, 5, respectively. The numbers at the bottom of the heat map represent the mouse identification numbers.

**Fig 6:**
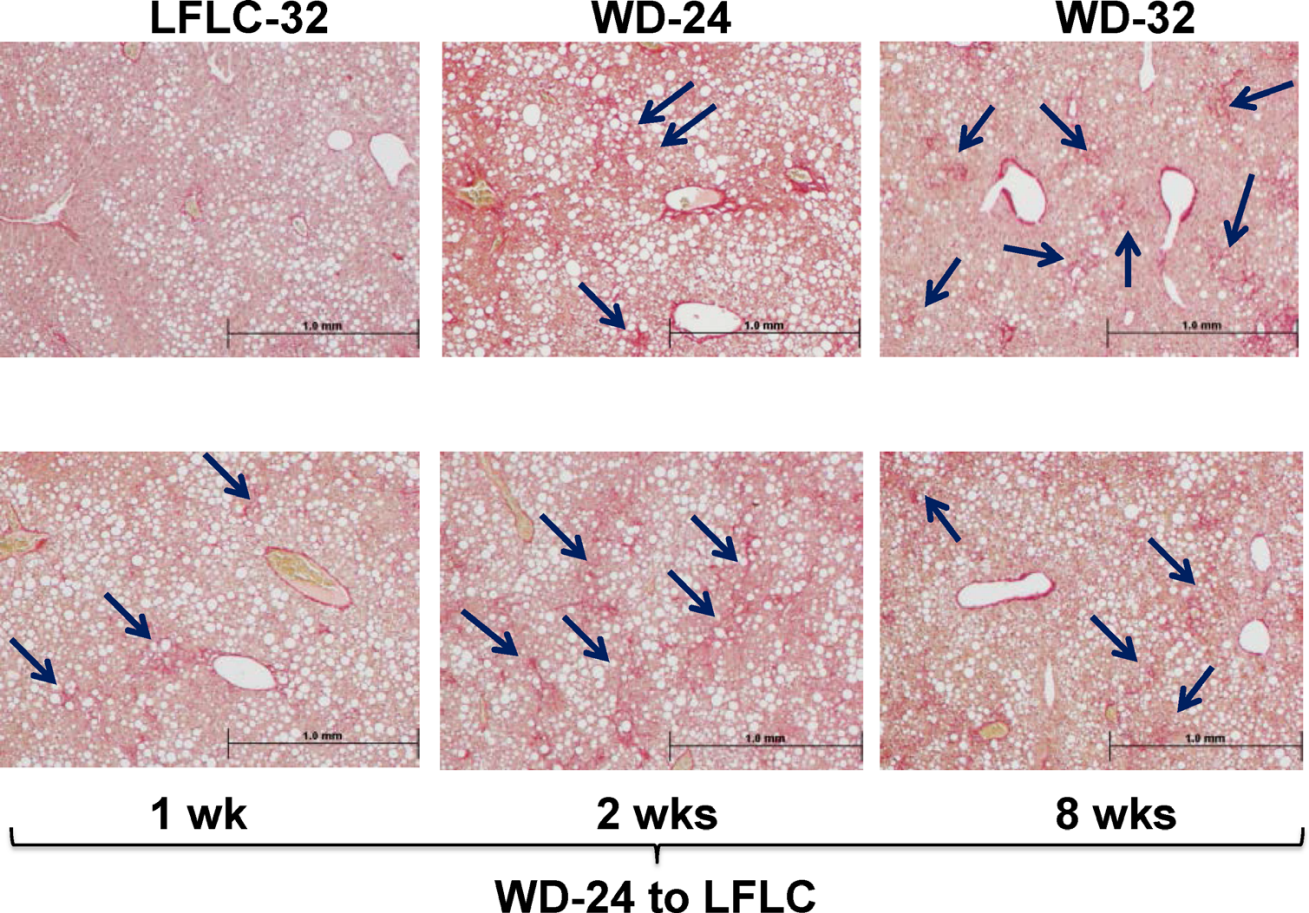
Time course analysis of hepatic histology of mice fed the LFLC or WD diets. Liver sections were stained with Sirius Red to reveal fibrosis appearing as red branching fibrosis (black arrows). The stained sections are from mice fed the WD for 24 weeks, the LFLC or WD for 32 weeks and mice fed the WD for 24 weeks and switched to the LFLC diet for 1, 2 or 8 weeks. The stained liver sections are representative of all livers in each group. The magnification of all images is 4X.

**Fig 7:**
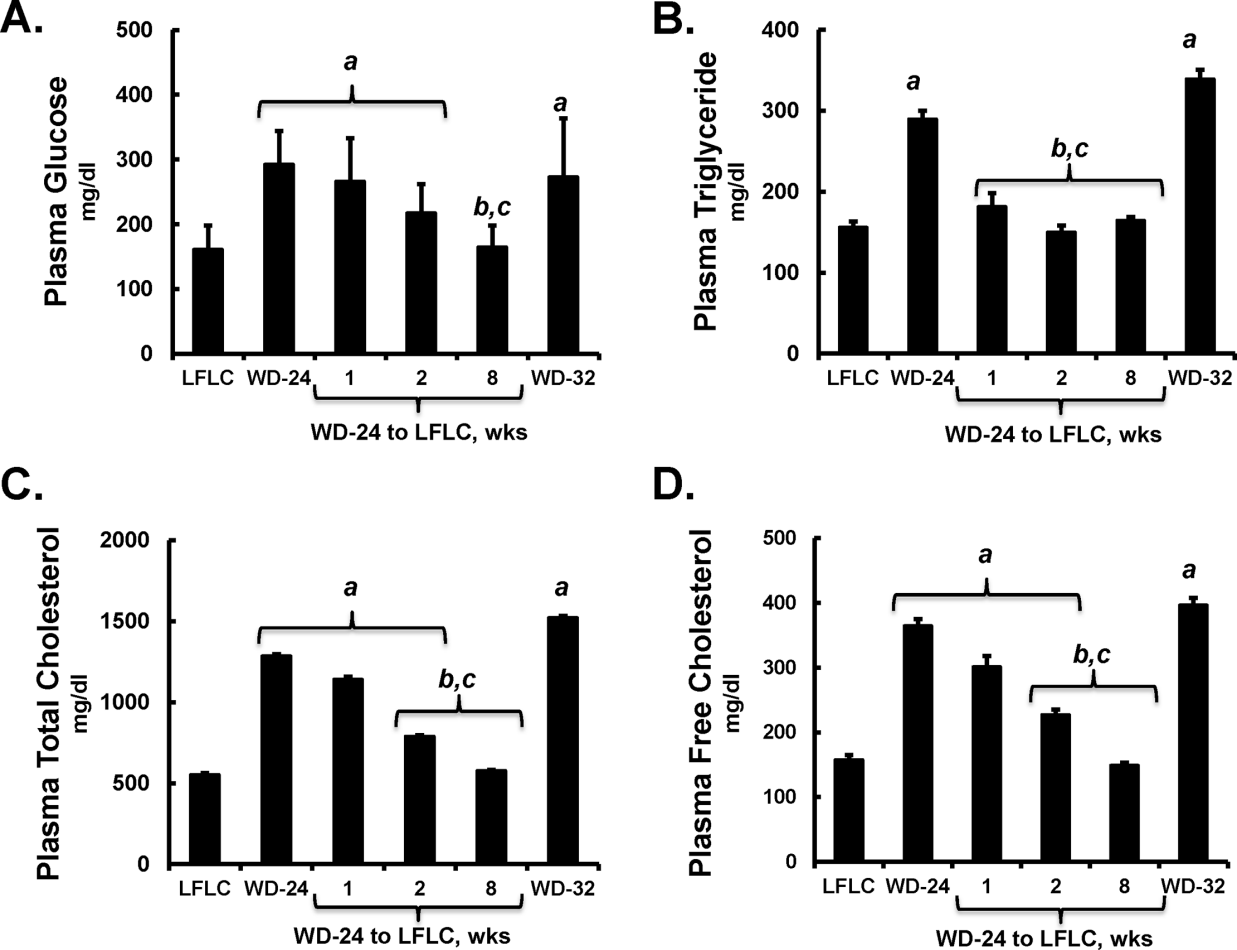
Time course of effects of diet on plasma markers of MetS. Plasma levels of glucose **[A],** triglyceride **[B],** total cholesterol **[C]** and free cholesterol **[D]** were assayed as described in Materials and Methods. Results are represented as plasma values; mean ± SD with 5–6 animals/group. ANOVA: **a**, p≤0.05 versus the LFLC group; **b**, p≤0.05 versus the WD-32 group; **c**, p≤0.05 versus the WD-24 group.

**Fig 8:**
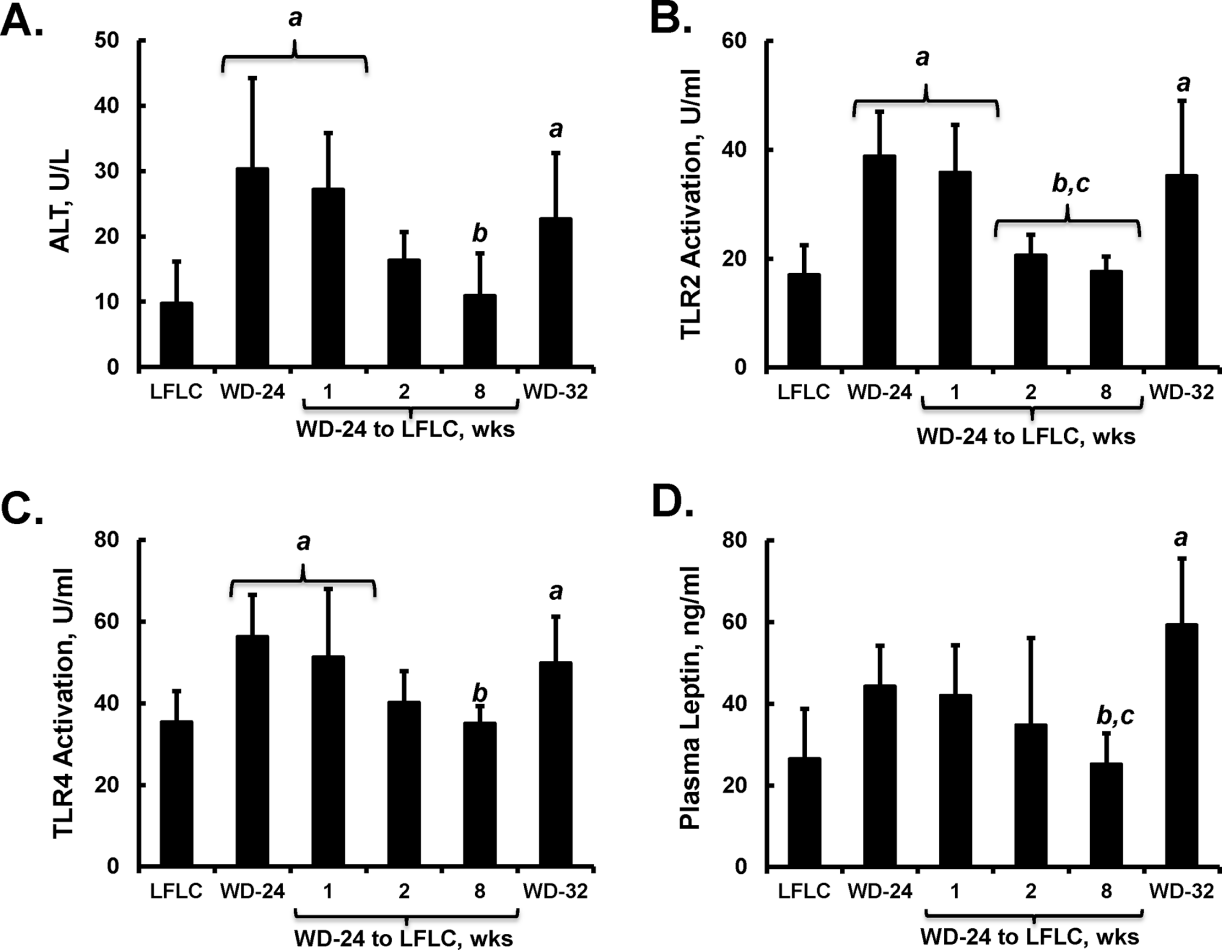
Time course of effect of diet on plasma markers of hepatic damage, inflammation and leptin. Plasma levels of alanine aminotransferase (ALT) **[A]**, activators of toll-like receptor 2 (TRL2), **[B]** and TLR4 **[C]**, and leptin **[D]** were quantified as described in Materials and Methods. Adiponectin was also quantified, but adiponectin was not significantly affected by diet (not shown). Results are represented as plasma values; mean ± SD with 5–6 animals/group. ANOVA: **a**, p≤0.05 versus the LFLC group; **b**, p≤0.05 versus the WD-32 group; **c**, p≤0.05 versus the WD-24 group.

**Fig 9:**
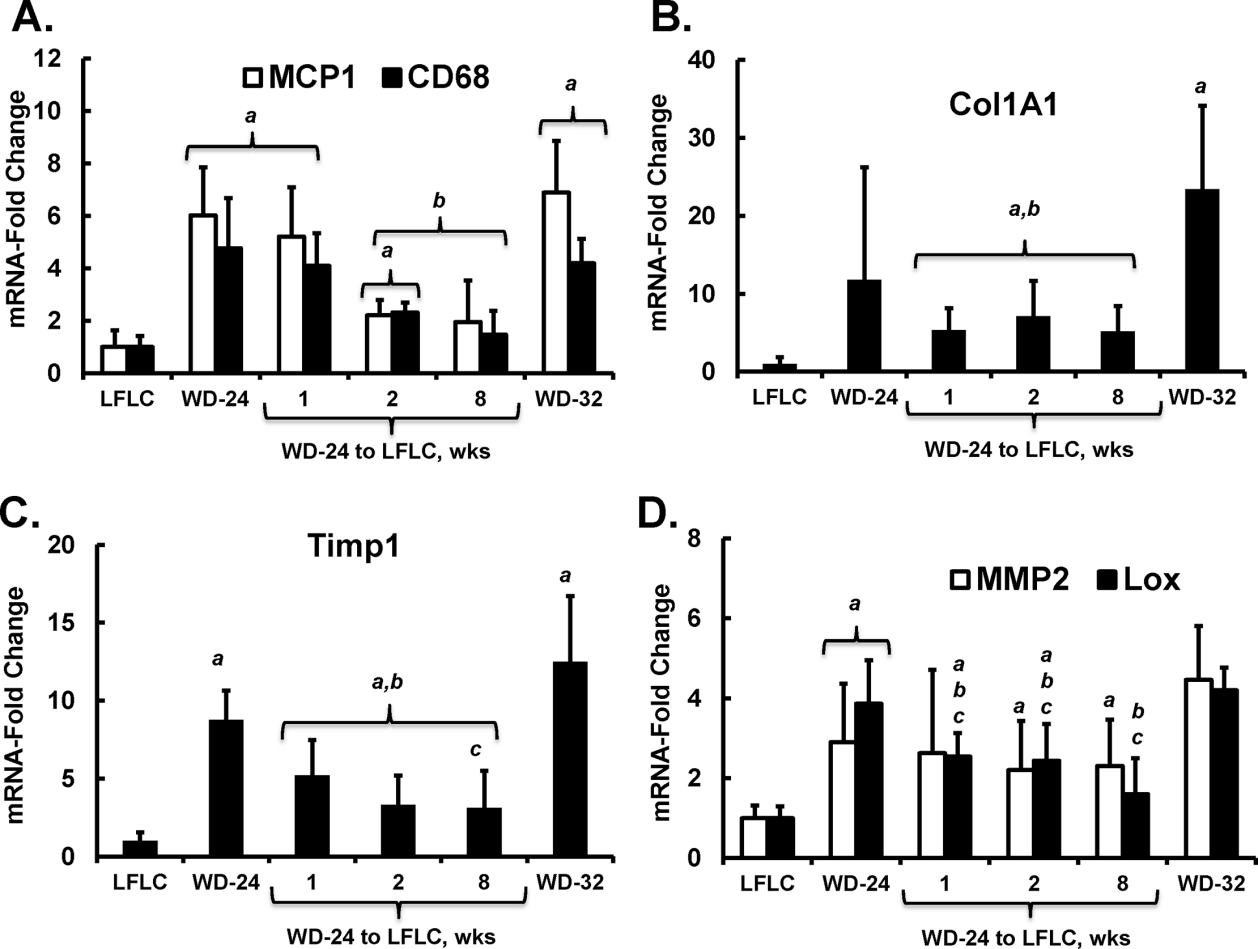
Time course of diet effects on hepatic gene expression markers of inflammation and fibrosis. Hepatic expression of mRNA transcripts encoding proteins involved in inflammation (*Mcp1*, *CD68*) **[A]** and fibrosis (*Col1A1* **[B]**, *Timp1* **[C]**, *Mmp2* and *Lox* **[D]**) were quantified by qRTPCR as described in Materials and Methods. Results are represented as mRNA-Fold Change; mean ± SD with 5–6 animals/group. ANOVA: **a**, p≤0.05 versus the LFLC group; **b**, p≤0.05 versus the WD-32 group; **c**, p≤0.05 versus the WD-24 group.

**Fig 10:**
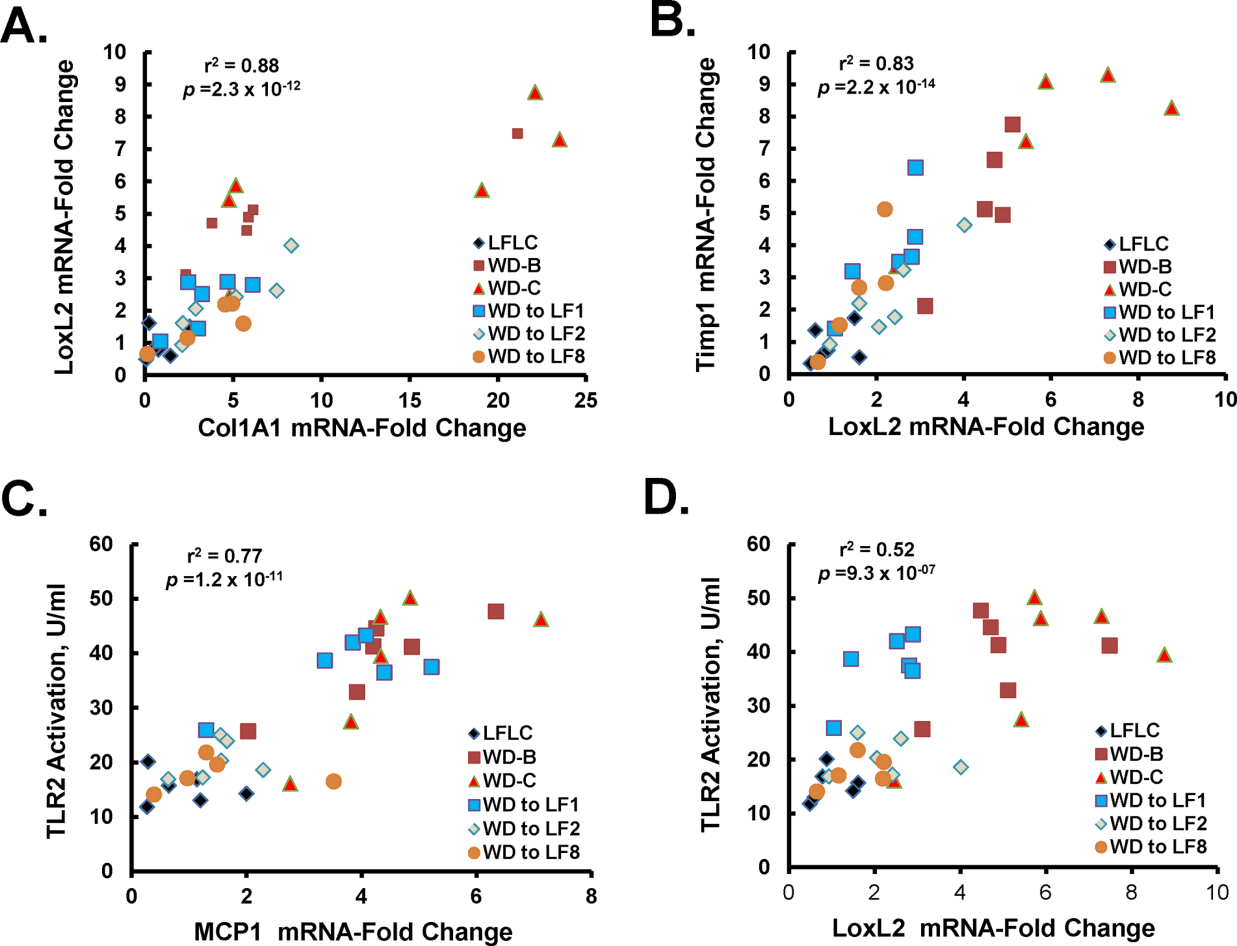
Regression analysis of plasma and hepatic markers of inflammation and fibrosis. Linear regression analysis was used to assess the association between hepatic transcripts involved in hepatic fibrosis, **[A]**: Col1A1 versus LoxL2; **[B]**: LoxL2 versus Timp1. This analysis was used to assess the association between plasma TLR2 activators and hepatic transcripts involved in inflammation **[C]**: *Mcp1* versus TLR2] and fibrosis **[D]**: *LoxL2* versus TLR2]. As indicated in Table 1, plasma TLR 2 activators were identified as a highly significant plasma marker of NASH. R^2^ and p-values were as described in Materials and Methods.

Having established that the LFLC diet can at least partially reverse many of the WD-induced features associated with NASH phenotype, we next determined the time course across which the LFLC diet promotes NASH remission. An overview of these effects is seen in the heat map (**Fig 5**), hepatic histology (**Fig 6**) and **Figs 7–10**where we quantify selected features. Mice fed the LFLC diet have a moderate level of steatosis and no detectable fibrosis (**Fig 6**). Mice fed the WD for 24 or 32 wks showed a higher level of steatosis (neutral lipid storage) and mild to moderate fibrosis (Sirius red stain branching fibrosis). Switching mice to the LFLC diet for up to 8 wks did not reverse hepatic steatosis or fibrosis (**Figs 3 & 6**).

### The LFLC diet promotes remission of plasma markers of NASH

Fasting plasma glucose was increased by feeding mice the WD and returned to control (LFLC) levels by 8 wks (**Fig 7**). Changes in plasma glucose paralleled diet-induced changes in plasma total and free cholesterol. Plasma triglycerides, in contrast, were induced by the WD, but return to control levels rapidly (within 1 wk) after the switch from the WD to the LFLC diet.

Plasma ALT was elevated (3-fold) by the WD and this marker of hepatic damage decreased to control levels within 2 wks following the switch to the LFLC diet (**Fig 8**). Plasma TLR2 and TLR4 activators represent both gut-derived microbial products and endogenously derived molecules that have been linked to systemic and hepatic inflammation and NASH [20–23]. Plasma TLR2 and TLR4 activators were increased by the WD and were decreased to control levels after 2 wks of feeding LFLC diet. Leptin, a cytokine primarily produced by adipose tissue, has been linked to hepatic fibrosis [24]. Plasma leptin was significantly elevated in the WD-32 group only.

### The LFLC diet promotes remission of hepatic gene expression markers associated with NASH

Analysis of hepatic gene expression provides evidence for diet effects on signaling pathways affecting inflammation, fibrosis, and oxidative stress. Feeding WD increased expression of *Mcp1*, *CD68* (**Fig 9**) and other markers of hepatic inflammation (**Fig 4**). Switching mice from the WD to the LFLC diet decreased hepatic abundance of these transcripts within 2 wks. This response paralleled changes in hepatic TLR2 and TLR4 activators (**Figs 8 & 9**).

The WD also increased expression of transcripts associated with hepatic fibrosis and matrix remodeling. Collagen 1A1 (*Col1A1*) is the major hepatic collagen subtype associated with NASH in rodents (**Fig 9**) and humans [25, 26]. *Col1A1* expression was highly variable in the WD-24 group, while the WD-32 group showed at >20-fold increase in *Col1A1* expression when compared to mice fed the LFLC diet for 32 wks. Switching WD-24 mice to LFLC for eight wks failed to return *Col1A1* expression to levels seen in control mice, a finding that agreed with the histology reported in **Fig 6**. Tissue-inhibitor of metalloprotease-1 (*Timp1*) expression was well induced in mice fed the WD for 24 or 32 wks. *Timp1* expression showed a similar overall profile as *Col1A1*, but with less variability in its expression. Metalloprotease-2 (*Mmp2*) and lysyl oxidase (*Lox*) are involved in ECM remodeling and collagen crosslinking, respectively. Both transcripts were elevated in the WD-24 and WD-32 groups. *Lox* expression returned to control levels after feeding LFLC for 8 wks. In contrast, *Mmp2* expression failed to return to control levels within 8 wks of switching from the WD to the LFLC diet.

### Regression Analyses

Regression analysis was used to assess associations between plasma and hepatic parameters. Statistical analysis ranked plasma and hepatic features associated with NASH in *Ldlr*^*-/-*^ mice; the top 10 features are listed in **Table 1**and all features that changed significantly are listed in **S5 Table**. The top plasma and hepatic features were TLR2 activators and lysyl oxidase-like-2 (*LoxL2*) mRNA, respectively. Regression analysis was used to determine the degree to which plasma and hepatic measures exhibited a similar expression patterns across the time course study. Hepatic genes associated with collagen production and catabolism (*Col1A1* & *Timp1)* were strongly associated with *LoxL2* expression (r^2^ ≥ 0.8) **(Fig 10A & 10B)**. *LoxL2* is one of several lysyl oxidases/lysyl oxidase-like enzymes involved in collagen cross-linking.

**Table 1:** Top 10 features associated with NASH in *Ldlr*^*-/-*^ mice as determined by ANOVA-Tukey HSD^1^.

Feature	p-value	Effect of WD	Function
Plasma TLR2 Activators	3.7 x 10^−10^	Increase	Promotes Inflammation
*LoxL2*	6.0 x 10^−10^	Increase	Collagen Crosslinking
Plasma Triglycerides	1.7 x 10^−9^	Increase	Dyslipidemia Marker
*Nox2*	8.0 x 10^−9^	Increase	Cellular Oxidative Stress
*Timp1*	2.3 x 10^−8^	Increase	Inhibits ECM Degradation
Plasma Free Cholesterol	2.5 x 10^−8^	Increase	Dyslipidemia Marker
20:0	1.2 x 10^−7^	Increase	Long Chain Saturated Fatty Acid
18:2,ω6	2.5 x 10^−7^	Decrease	Essential Fatty Acid
*Hmox1*	2.3 x 10^−7^	Increase	Anti-Oxidant Defense
18:3,ω6	2.5 x 10^−7^	Increase	Long Chain PUFA

^1^All data used to construct the heat map in Fig 5 was used for statistical analysis using the MetaboAnalyst 3.0 statistical package. The table above lists the top 10 features with the lowest p-values, i.e., high significance. The complete list of features and their p-values is provided as “**S5 Table**. Significant features associated with NASH in *Ldlr*^*-/-*^ mice as determined by ANOVA-Tukey HSD”.

TLR2 agonists bind TLR2 receptors and activate NFκB, a key transcription factor involved in inflammation [27]. Hepatic *Mcp1* is a NFκB target gene; plasma TLR2 agonist levels were strongly associated with hepatic *Mcp1* gene expression (r^2^ = 0.77) **(Fig 10C).** The association between TLR2 agonist levels and expression of LoxL2 was also strong (r^2^ = 0.52) **(Fig 10D)**.

Levels of plasma lipids (triglyceride and cholesterol) exhibited a modest association with hepatic expression of *LoxL2* and *Timp1* (not shown). Interestingly, the association between plasma TLR2 agonist and hepatic lipids was stronger with hepatic cholesterol (r^2^ = 0.19) than triglyceride (r^2^ = 0.025). Similarly, the association between hepatic *LoxL2* expression was stronger with hepatic cholesterol r^2^ = 0.35 than hepatic triglyceride (r^2^ = 0.063). The associations between hepatic triglycerides and TLR2 agonist or *LoxL2* expression, however, were weak.

### Association between hepatic PUFA and NASH markers

**Fig 11:**
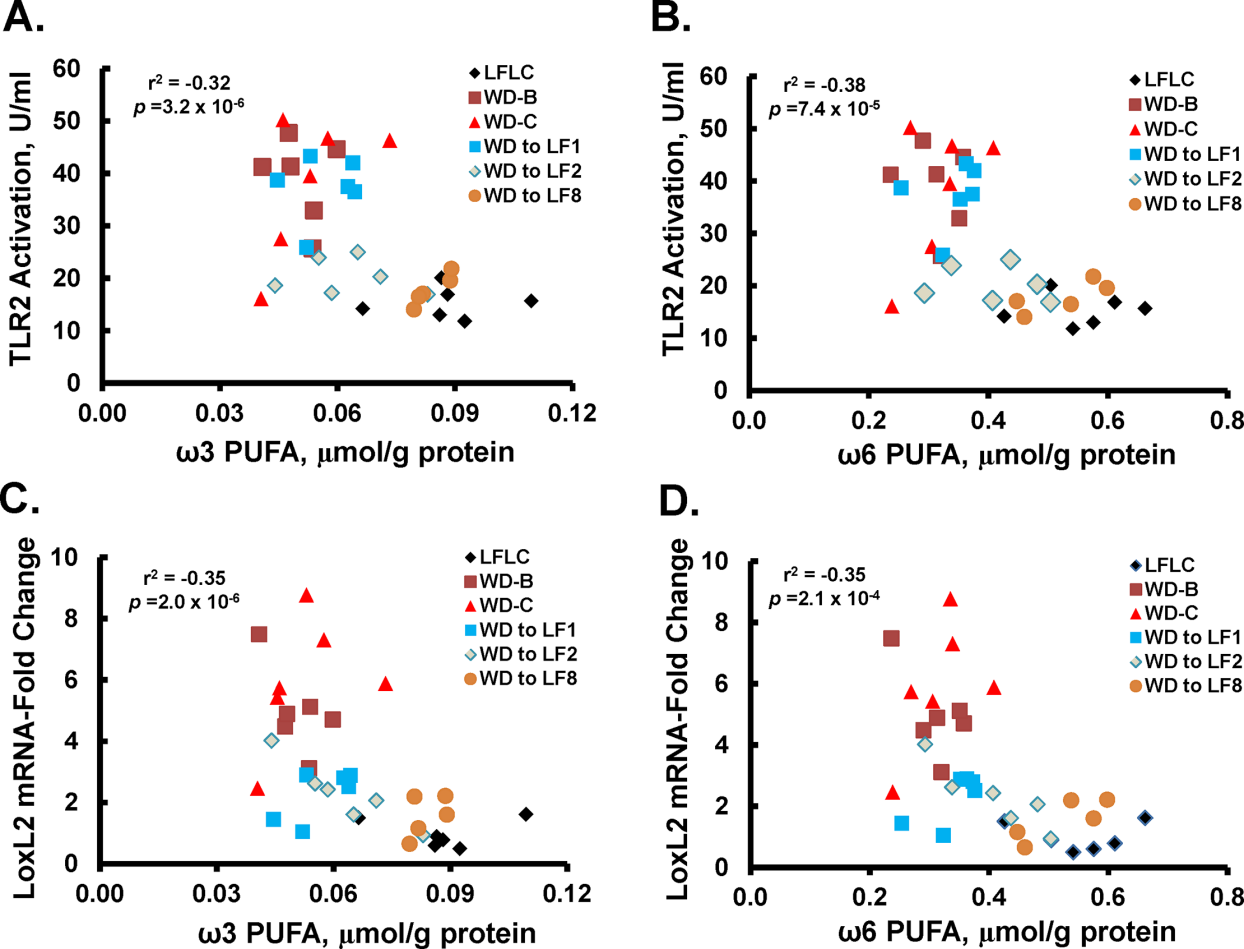
Regression analysis of markers of inflammation, fibrosis and hepatic PUFA. Linear regression analysis was used to assess the association between hepatic PUFA and plasma TLR2 activation **[A]**: ω3 PUFA versus TLR2 activation; **[B]**: ω6 PUFA versus TLR2 Activation] and hepatic *LoxL2* transcript abundance **[C]**: ω3 PUFA versus *LoxL2* mRNA; **[D]**: ω6 PUFA versus *LoxL2* mRNA]. R^2^ and p-values were derived as described in Materials and Methods.

**Fig 12:**
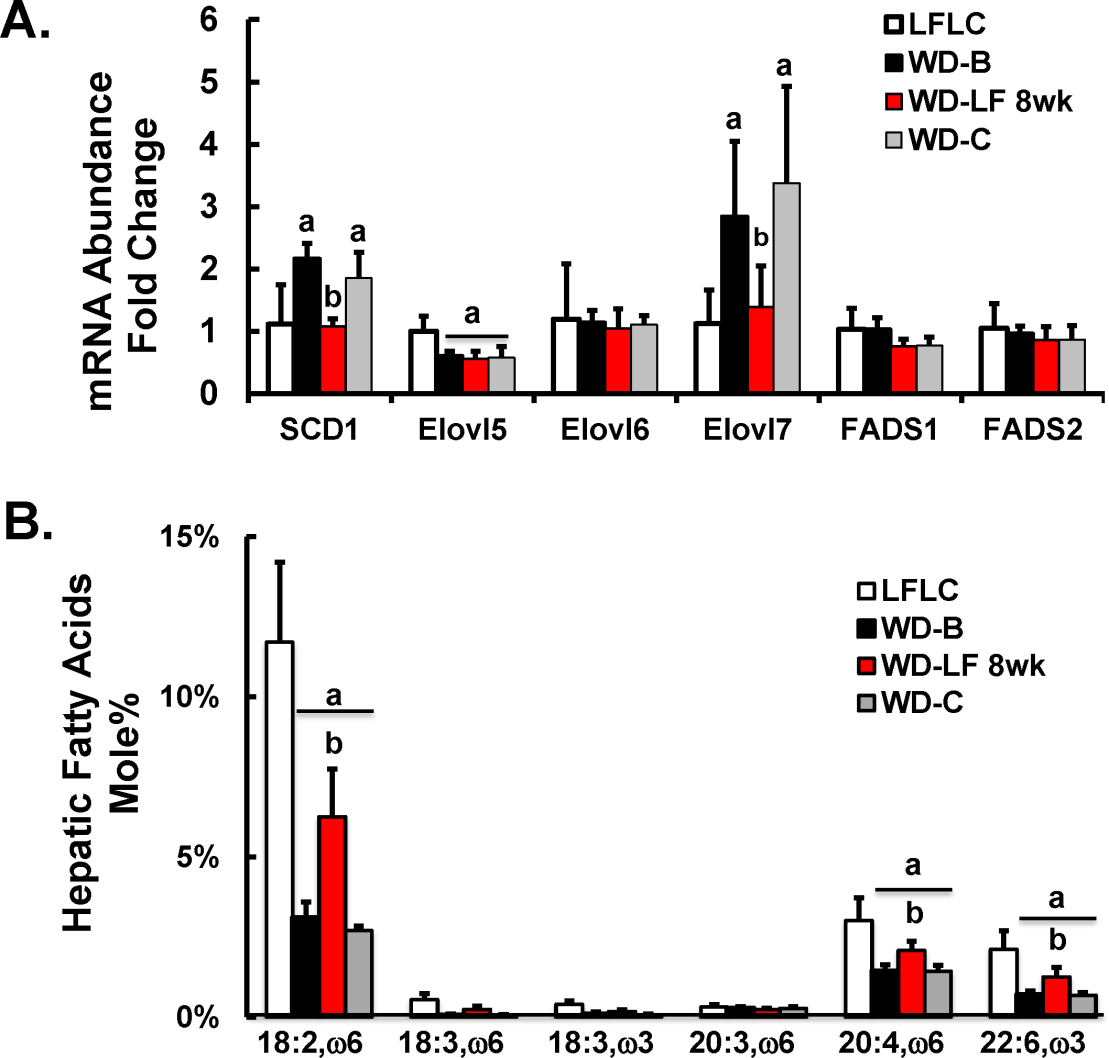
Diet effects on the hepatic expression of enzymes involved in desaturation and elongation of saturated, mono- and polyunsaturated fatty acids. **[A]**: Hepatic transcript abundance of enzymes was quantified as described in Materials and Methods. Results are represented as mRNA-Fold Change; mean ± SD with 5–6 animals/group. **[B]**: Hepatic PUFA content. Total hepatic fatty acids were quantified as described in Materials and Methods. Hepatic PUFA are represented as Fatty Acid Mole%, mean ± SD with 5–6 animals/group. ANOVA: **a**, p≤0.05 versus the LFLC group; **b**, p≤0.05 versus the WD-32 group.

Humans with NASH and *Ldlr*^*-/-*^ mice fed the WD have lower plasma and hepatic content of ω3 and ω6 PUFA [14,15, 28–30]. Both studies reported here (**Figs 2 & 5**) demonstrate that feeding mice the WD decreased hepatic ω3 and ω6 PUFA and that returning WD-fed mice to either a NP or LFLC diet, at least partially, restored hepatic levels of these PUFA. Herein, we show that there is a modest inverse association between changes in hepatic PUFA and plasma and hepatic markers of NASH, i.e., TLR2 agonist and *LoxL2* mRNA (r^2^ = 0.32 and 0.35) respectively (**Fig 11**). WD feeding altered expression for some genes involved in fatty acid desaturation (*Scd1*) and elongation (*Elovl5 & Elovl7*) (**Fig 12**). Specifically, WD increased the expression of *Scd1* and *Elovl7*, suppressed *Elovl5* expression and had no effect on *Fads1*, *Fads2* or *Elov6* expression. Switching mice from the WD to the LFLC for 8 wks returned *Scd1* and *Elovl7* gene expression to levels seen in mice fed the LFLC diet for 32 wks. *Elovl5*, in contrast, was not restored to normal levels by the LFLC diet. Overall, changes in hepatic desaturases and elongases do not fully explain WD-induced changes in hepatic PUFA content.

## Discussion

Our goal was to evaluate the capacity of two low-fat low-cholesterol diets to reverse WD-induced MetS and NASH in male *Ldlr*^*-/-*^ mice. Both non-purified chow (**NP**) and purified (low-fat low-cholesterol, **LFLC**) diets were low in fat (13% and 10%, respectively) and low in cholesterol (0.014% and 0.0014%, respectively). The defining macronutrient difference between the two diets was the sucrose content (5.5% versus 35% total calories) in the NP and LFLC diets, respectively. This difference in simple sugar content of the diet is, at least partially, responsible for the differences in outcomes between the studies. While we recognize that there are small differences in study duration (29 wks total for Study 1 and 32 wks total for Study 2) as well as mouse ages when starting the study (9 versus 10 wks of age), the outcomes and conclusions of the studies are strikingly similar.

In contrast to the NP diet, the LFLC diet failed to reverse WD induced hepatosteatosis (Figs 1, 3 & 6). Moreover, both the NP and LFLC diets had limited capacity to reverse WD-induced hepatic fibrosis as measured biochemically and histologically. Both diets also failed to restore WD-induced alterations in hepatic content of specific MUFA and PUFA. The outcome of these studies establish that the composition of the diet used to promote NASH/fibrosis remission significantly impacts the degree to which NASH remission occurs and that all features of NASH are not reversible on the same time scale. These results also suggest that interventions, in addition to weight loss and the consumption of a low-fat low-cholesterol diet, are required to fully reverse WD induced NASH.

### Metabolic plasma parameters

Feeding *Ldlr*^*-/-*^ mice the WD elevates plasma parameters associated with NASH and MetS, including plasma lipids (triglycerides and cholesterol) and glucose (**Figs 2, 4, 5 and 7**). Changes in these metabolic markers paralleled changes in circulating biomarkers of liver injury, e.g., ALT (**Fig 8**). In humans with NASH and T2DM, weight loss results in decreased levels of these plasma markers [31]. Returning WD-fed mice to control diets (NP or LFLC) lowered all plasma metabolic markers to normal. These plasma metabolic parameters decrease rapidly following the change in diet and precede changes in the overall disease status of the NASH liver and therefore these circulating biomarkers of inflammation, glucose homeostasis, and liver injury do not parallel hepatic status with respect to steatosis or fibrosis.

### Inflammation

Both systemic and hepatic inflammation can drive NASH progression [15, 30, 32]. WD feeding increased plasma inflammatory markers (TLR2 & TLR4 agonists, leptin) and induced hepatic mRNA transcripts linked to inflammation. TLR2 and TLR4 bind bacterial components as well as some host components (oxidized lipids, extracellular matrix components) [33–36]. Our studies demonstrate that extended WD feeding increased both TLR2 and TLR4 plasma agonists. Activation of TLR2 & TLR4 signaling pathways involves increasing NFκB nuclear translocation and subsequent upregulation of inflammatory genes including signaling cytokines, such as TNFα and IL1β. Gut derived signals, such as lipopolysaccharide (**LPS**/endotoxin), activate TLRs and contribute to the pathogenesis of NASH [27, 37–40]. Blood levels of LPS are increased in both humans [41] and mice [15] with NAFLD. High-fat diets (such as the WD) promote increases in circulating LPS by moving LPS from the gut lumen to the circulation via chylomicron assembly and transport [38, 42]. Taken together, our data suggests that the WD is a potent inducer of systemic inflammation that contributes to the pathogenesis of NASH. Providing an alternative diet (LFLC or NP) to mice can ameliorate some of these systemic inflammatory mediators. The time course of remission of these indicators suggests that hepatic expression of inflammatory genes (*Mcp1* and *CD68*) decreased on the same time-scale as the loss of the plasma TLR2 & TLR4 activators (**Figs 8 & 9**).

### Reversibility of diet-induced hepatic fibrosis

A key goal of these studies was to determine the capacity of dietary modification to reverse WD-induced hepatic fibrosis. A recent study identified fibrosis stage as the driving predictor of overall and disease-specific mortality in patients with NASH [43]; thus knowing whether fibrosis is reversible is necessary in order to address treatment. The reversibility of hepatic fibrosis has been previously studied in models of acute chemically (carbon tetrachloride, **CCL4**) induced fibrosis [5] and bile duct ligation (**BDL**) [7]. The reversal of CCL4 induced fibrosis is rapid, i.e., full resolution of fibrosis occurred within 30 days [6]. Hepatic fibrosis resolution in the BDL model required more than 12 weeks after the stimulus was removed [7]. However, neither of these approaches recapitulates the onset or remission of hepatic fibrosis in the context of MetS and/or type 2 diabetes. Therefore, our studies focused on a model of WD-induced NASH and fibrosis, which is a major pathophysiological feature associated with NASH in obese humans.

WD-feeding for 22–32 wks greatly elevated biochemical and histological markers of fibrosis. Returning mice to diets low in fat and cholesterol reduced, but did not resolve hepatic fibrosis (**Figs 1, 3 & 6**). The reversibility of NASH in the CCL4 and BDL models suggests that hepatic fibrosis is reversible upon removal of the factor(s) stimulating the development and progression of NASH. Given that control mice on the LFLC diet display little fibrosis this suggests that our intervention of switching from WD to LFLC should have reversed hepatic fibrosis. However, mice that were switched to NP or LFLC still displayed fibrosis following 8 wks on these low-fat, low-cholesterol diets. Taken together this data suggests that fibrosis that develops from long term WD-feeding (i.e., within the pathophysiological context of MetS) is not readily reversible by dietary modification alone; and full reversal of fibrosis will required other strategies.

The fibrotic status of the liver is governed by the balance of ECM deposition and turnover; and this process is controlled by the expression of ECM components (collagens, elastins, etc.), Mmps, Timps, and proteins controlling collagen cross-linking (lysyl oxidase [Lox] and LoxL subtypes 1–4 [5, 44–48]. WD-induced hepatic fibrosis in *Ldlr*^*-/-*^ mice involves the induction of collagen (Col1A1, Col1A2, Col4A1) production. This increase in collagen subtypes expression concurs with data in humans [49]. In addition, the WD also induces several Mmps, Timps and enzymes involved in chemical cross-linking (Lox & LoxL subtypes) [25]. The biochemical and histological evidence of fibrosis in WD fed mice we report provides clear evidence that the rate of collagen production exceeds its degradation. Our analysis of fibrosis (**Figs 1, 3 and 6**) is in agreement with findings in humans following weight loss after gastric surgery (gastric band or Roux-en-Y) [8, 31, 50]. Unfortunately in humans, there are both significant long-term complications and high failure rates of gastric surgeries [51] and thus these approaches may not be ideal for addressing both obesity and NASH.

### Hepatic Cholesterol Content

While dietary cholesterol is a well-known driver of NASH progression [52], dysregulated cholesterol synthesis and metabolism is also a feature of NASH [53]. Cholesterol can be converted to oxysterols, some of which are ligands for LXR, a key regulator of lipogenic gene expression [54]. We and others have reported that hepatic cholesterol content is associated with NASH severity [14, 15, 52]. Despite the very low cholesterol content in the LFLC diet (**S1 Table**), livers in the WD-LFLC-8 wk group exhibited no reduction in hepatic cholesterol or triglyceride when compared to the WD-32 wk group (**S3 Table**). This suggests that removal of excess cholesterol from the diet is not sufficient to decrease hepatic cholesterol content after the initial induction of NASH. Although livers of WD-LFLC-8 wk mice had 2- to 3-fold higher levels of cholesterol, most of the hepatic inflammation markers were resolved (**Fig 9**). These findings reveal dissociation between hepatic cholesterol content and inflammation.

The role of simple sugars in the development of fatty liver has been previously described [55, 56]. Excess dietary sucrose as seen in the WD and LFLC diets likely increases both *de novo* lipogenesis and cholesterol synthesis leading to increased formation of lipid as triglycerides and cholesterol esters and their storage in lipid droplets, i.e., hepatosteatosis, as is seen in the WD and WD-LFLC-8wk groups (**Fig 3**). Feeding mice the NP diet after NASH induction (WD-NP group), lowered both dietary cholesterol and simple sugars, and resolved hepatosteatosis; both triglycerides and cholesterol in the liver returned to normal (**S2 Table**). Since hepatic inflammation resolved in both the WD-NP and WD-LFLC groups, stored cholesterol, i.e., cholesterol ester, is not harmful in and of itself. However, reducing hepatic cholesterol requires lowering both dietary cholesterol and simple sugar.

### Hepatic Fat Content

In contrast to fibrosis, the reversal of diet-induced hepatosteatosis is significantly affected by the type of diet used to promote remission. The NP diet, which is low in fat, cholesterol and simple sugar, promoted greater reduction in hepatosteatosis than the LFLC diet, a diet with higher levels of sucrose **(S2 and S3 Tables).** The sucrose content in the LFLC diet is comparable to the sucrose content in the WD. The finding that the LFLC diet failed to reverse hepatosteatosis strongly suggests that limitation of dietary simple sugar in addition to dietary fats is required to reverse hepatosteatosis. The role of fructose and other simple sugars is well established in promoting neutral lipid storage in the liver [57]. However, the finding that some of the control mice fed LFLC exhibited histologically mild hepatosteatosis (biochemically, triglyceride measures between LFLC and NP are not different) but not NASH supports the concept that other factors, such as systemic inflammatory factors, contribute to NASH progression.

While most of the WD induced alterations in liver and plasma profiles were reduced by removal of WD feeding, notably, hepatic fatty acid profiles were not reversed to levels seen in control mice. Specifically, WD feeding alters the distribution of fatty acids by increasing MUFAs and decreasing **ω**3 and **ω**6 PUFA (**Figs 2, 4, 5 & 12**). In particular, WD feeding increased the hepatic content of oleic acid (18:1,ω9) and cis-vaccenic acid (18:1,ω7). While 18:1,ω9 is found in the diet, 18:1,ω7 is not in the NP, LFLC or WD diets [15, 16]. As such, 18:1,ω7 is generated by the elongation of 16:1,ω7; and this fatty is generated by SCD1-mediated desaturation of palmitic acid (16:0). The increase in hepatic MUFA (16:1,ω7, 18:1,ω7, 18:1,ω9) were associated with corresponding increases in *SCD1* and *Elovl7* expression (**Fig 12**).

We also report that the WD decreased hepatic content of linoleic acid (18:2,ω6), α-linolenic acid (18:3,ω3), arachidonic acid (20:4,ω6) and docosahexaenoic acid (22:6,ω3). All diets are essential (18:2,ω6 and 18:3,ω3) fatty acid sufficient. The decline in the C_20-22_ ω3 and ω6 PUFA was associated with decreased *Elovl5* expression, but no change in *Fads1* or *Fads2* expression (**Fig 12**). An alternative explanation for the decline in hepatic C_20-22_ PUFA abundance may be due to substrate competition at the level of *Fads2*. *Fads2* was recently reported to desaturate saturated fatty acids like palmitate [58] and there is an abundance of palmitate in fatty livers [15].

Placing WD fed mice on the LFLC diet for 8 wks partially restored ω3 and ω6 PUFA content to levels seen in mice fed the NP or LFLC diets for the duration of the study. We previously reported that increasing dietary C_20-22_ ω3 PUFA, particularly DHA, in the WD attenuated WD-induced NASH in *Ldlr*^*-/-*^ mice. DHA was more effective than EPA at preventing hepatosteatosis, inflammation and fibrosis [15]. Dietary DHA increased hepatic levels of both DHA and EPA (via retro-conversion) and interfered with TGFβ signaling in the liver [25]. TGFβ plays a major role in the onset and progression of fibrosis [22].

Finally, our analyses revealed a weak association between top NASH features (i.e. *LoxL2* mRNA and plasma TLR2 agonists) and total hepatic triglyceride (r^2^ = 0.063 and r^2^ = 0.025). In contrast, these top NASH features showed a stronger association with hepatic ω3 and ω6 fatty acid content (r^2^ = 0.32 and r^2^ = 0.35) (**Fig 11**). These finding suggest that the degree of NASH may be more related to the hepatic content of specific fatty acids, rather than the total amount of hepatic triglyceride.

## Conclusions

Long-term WD feeding was used to induce NASH in *Ldlr*^*-/-*^ mice. We tested the efficacy of two low-fat, low-cholesterol diets to promote NASH remission. While most gene expression markers, all plasma parameters examined, and body weights returned to normal, this intervention failed to reverse both hepatic fatty acid profiles and NASH-associated fibrosis. Based on these studies, removing WD and switching to low fat-low cholesterol (NP or LFLC) diets is not sufficient to allow for full resolution of NASH. These studies also demonstrated a differential response of NASH remission dependent upon the diet used. The NP diet was superior to the LFLC diet in resolving NASH. This suggests that the beneficial results of these diets are not solely based on removal of fat and cholesterol from the diet, but may also depend on the level of dietary simple sugar, i.e., sucrose and fructose. Whether full resolution, and in particular, removal of fibrosis occurs with continued weight maintenance, addition of exercise, or targeted drug therapies coupled with lifestyle changes is yet to be determined.

## Supporting Information

S1 Fig Effect of the NP and WD on hepatic gene expression-Study1. Mice were fed the Chow or WD diets as described in Materials and Methods. RNA was extracted and the abundance of mRNA transcripts encoding proteins involved in inflammation [**A**], oxidative stress [**B**] and fibrosis and ECM remodeling [**C**] were quantified. Results are presented as mRNA Abundance-Fold Change. Results are expressed as mean ± SD with 4 animals/group. **a**, p≤0.05 versus the chow group.
(TIFF)

S2 Fig Effect of diet on hepatic fatty acid composition-Study 1. Hepatic lipids were extracted, saponified, converted to fatty acid methyl esters and quantified by gas chromatography as described in Material and Methods. [**A]:** Hepatic fatty acid content, results are expressed as nmol/mg protein; [**B]:** Sum of fatty acids in specific classes; results are expressed as nmol/mg protein; **[C]:** Hepatic C_20-22_ n-3 and n-6 fatty acids, results are expressed as nmol/mg protein. Results are expressed as mean ± SD with 4 animals/group. **a**, p≤0.05 versus the chow group.
(TIFF)

S1 Table Diet Composition. (DOCX)

S2 Table Body weight, plasma and liver parameters-Study 1. (DOCX)

S3 Table Body weight, plasma and liver parameters-Study 2. (DOCX)

S4 Table Comparison of NP- versus LFLC-fed *Ldlr*^*-/-*^ mice. (DOCX)

S5 Table Significant features associated with NASH in *Ldlr*^*-/-*^ mice as determined by ANOVA-Tukey HSD. (DOCX)

## References

[1] Cohen JC, Horton JD, Hobbs HH. Human fatty liver disease: old questions and new insights. Science. 2011;332(6037):1519–1523. 10.1126/science.1204265 21700865 PMC3229276

[2] Welsh JA, Karpen S, Vos MB. Increasing prevalence of nonalcoholic fatty liver disease among United States adolescents, 1988–1994 to 2007–2010. J Pediatr. 2013;162(3):496–500. e1. 10.1016/j.jpeds.2012.08.043 23084707 PMC3649872

[3] Vernon G, Baranova A, Younossi Z. Systematic review: the epidemiology and natural history of non-alcoholic fatty liver disease and non-alcoholic steatohepatitis in adults. Alimentary Pharmaclo Thera. 2011;34(3):274–285. 10.1111/j.1365-2036.2011.04724.x 21623852

[4] McCullough AJ. Epidemiology of the metabolic syndrome in the USA. J Dig Dis. 2011;12(5):333–340. 10.1111/j.1751-2980.2010.00469.x 21091931

[5] Fallowfield JA, Mizuno M, Kendall TJ, Constandinou CM, Benyon RC, Duffield JS, et al Scar-associated macrophages are a major source of hepatic matrix metalloproteinase-13 and facilitate the resolution of murine hepatic fibrosis. J Immuno. 2007;178(8):5288–5295. 10.4049/jimmunol.178.8.5288 17404313

[6] Troeger JS, Mederacke I, Gwak GY, Dapito DH, Mu X, Hsu CC, et al Deactivation of hepatic stellate cells during liver fibrosis resolution in mice. Gastroentero. 2012;143(4):1073–83. e22. 10.1053/j.gastro.2012.06.036 PMC3848328 22750464

[7] Popov Y, Sverdlov DY, Bhaskar KR, Sharma AK, Millonig G, Patsenker E, et al Macrophage-mediated phagocytosis of apoptotic cholangiocytes contributes to reversal of experimental biliary fibrosis. Am J Physiol-Gastrointest Liver Physio. 2010;298(3):G323–G334. 10.1152/ajpgi.00394.2009 PMC2838516 20056896

[8] Lassailly G, Caiazzo R, Buob D, Pigeyre M, Verkindt H, Labreuche J, et al Bariatric Surgery Reduces Features of Non-alcoholic Steatohepatitis in Morbidly Obese Patients. Gastroentero. 2015; 149(2): 379–388. 10.1053/j.gastro.2015.04.014 25917783

[9] Marcellin P, Asselah T. Long-term therapy for chronic hepatitis B: Hepatitis B virus DNA suppression leading to cirrhosis reversal. J Gastroentero Hepato. 2013;28(6):912–23. 10.1111/jgh.12213 23573915

[10] Iredale JP. Models of liver fibrosis: exploring the dynamic nature of inflammation and repair in a solid organ. J Clin Invest. 2007;117(3):539–548. 17332881 10.1172/JCI30542 PMC1804370

[11] Ramachandran P, Iredale J. Liver fibrosis: a bidirectional model of fibrogenesis and resolution. Qjm. 2012;105(9):813–817. 10.1093/qjmed/hcs069 22647759 PMC3424469

[12] Zois C, Baltayiannis G, Karayiannis P, Tsianos E. Systematic review: hepatic fibrosis–regression with therapy. Aliment Pharmacol Thera. 2008;28(10):1175–1187. 10.1111/j.1365-2036.2008.03840.x 18761707

[13] Bieghs V, Van Gorp PJ, Wouters K, Hendrikx T, Gijbels MJ, van Bilsen M, et al LDL receptor knock-out mice are a physiological model particularly vulnerable to study the onset of inflammation in non-alcoholic fatty liver disease. PLoS One. 2012;7(1):e30668 10.1371/journal.pone.0030668 22295101 PMC3266276

[14] Depner CM, Torres-Gonzalez M, Tripathy S, Milne G, Jump DB. Menhaden Oil Decreases High-Fat Diet–Induced Markers of Hepatic Damage, Steatosis, Inflammation, and Fibrosis in Obese *Ldlr*^*−/−*^ Mice. J Nutr. 2012;142(8):1495–1503. 10.3945/jn.112.158865 22739374 PMC3397337

[15] Depner CM, Philbrick KA, Jump DB. Docosahexaenoic acid attenuates hepatic inflammation, oxidative stress, and fibrosis without decreasing hepatosteatosis in a *Ldlr*^*−/−*^ mouse model of western diet-induced nonalcoholic steatohepatitis. J Nutr. 2013;143(3):315–323. 10.3945/jn.112.171322 23303872 PMC3713021

[16] Tripathy S, Torres-Gonzalez M, Jump DB. Elevated hepatic fatty acid elongase-5 activity corrects dietary fat-induced hyperglycemia in obese C57BL/6J mice. J Lipid Res. 2010;51(9):2642–2654. 10.1194/jlr.M006080 20488798 PMC2918446

[17] Jump DB, Torres-Gonzalez M, Olson LK. Soraphen A, an inhibitor of acetyl CoA carboxylase activity, interferes with fatty acid elongation. Biochem Pharmac. 2011;81(5):649–660. 10.1016/j.bcp.2010.12.014 PMC3031740 21184748

[18] Xia J, Sinelnikov IV, Han B, Wishart DS. MetaboAnalyst 3.0—making metabolomics more meaningful. Nucleic Acids Res. 2015: 43(W1):W251–W257. 10.1093/nar/gkv380 25897128 PMC4489235

[19] Tripathy S, Jump DB. Elovl5 regulates the mTORC2-Akt-FOXO1 pathway by controlling hepatic cis-vaccenic acid synthesis in diet-induced obese mice. J Lipid Res. 2013;54(1):71–84. 10.1194/jlr.M028787 23099444 PMC3520542

[20] Roh YS, Seki E. Toll-like receptors in alcoholic liver disease, non-alcoholic steatohepatitis and carcinogenesis. J Gastroentero Hepato. 2013;28(S1):38–42. 10.1111/jgh.12019 PMC3721430 23855294

[21] Seki E, De Minicis S, Österreicher CH, Kluwe J, Osawa Y, Brenner DA, et al TLR4 enhances TGF-β signaling and hepatic fibrosis. Nature Med. 2007;13(11):1324–1332. 17952090 10.1038/nm1663

[22] Schuppan D, Kim YO. Evolving therapies for liver fibrosis. J Clin Invest. 2013;123(5):1887–1901. 10.1172/JCI66028 23635787 PMC3635731

[23] Cengiz M, Ozenirler S, Elbeg S. The role of serum toll-like receptors 2 and 4 in nonalcoholic steatohepatitis and liver fibrosis. J Gastroentero Hepatol. 2015 30(7):1190–1196. 10.1111/jgh.12924 25684563

[24] Magkos F, Fabbrini E, Klein S. Pathogenesis of Hepatic Steatosis and Fibrosis: Role of Leptin. Leptin: Springer; 2015 p. 89–101.

[25] Lytle KA, Depner CM, Wong CP, Jump DB. Docosahexaenoic Acid Attenuates Western Diet Induced Hepatic Fibrosis in *Ldlr*^*-/-*^ Mice by Targeting the TGFβ-Smad3 Pathway. J Lipid Res. 2015; 56: 1936–1946 10.1194/jlr.M061275 26315048 PMC4583081

[26] Clapper JR, Hendricks MD, Gu G, Wittmer C, Dolman CS, Herich J, et al Diet-induced mouse model of fatty liver disease and nonalcoholic steatohepatitis reflecting clinical disease progression and methods of assessment. Am J Physiol Gastrointest Liver Physiol. 2013 10 1;305(7):G483–G495. 10.1152/ajpgi.00079.2013 23886860

[27] Yang R-B, Mark MR, Gray A, Huang A, Xie MH, Zhang M, et al Toll-like receptor-2 mediates lipopolysaccharide-induced cellular signalling. Nature. 1998;395(6699):284–288. 9751057 10.1038/26239

[28] Arendt BM, Comelli EM, Ma DW, Lou W, Teterina A, Kim T, et al Altered hepatic gene expression in nonalcoholic fatty liver disease is associated with lower hepatic n-3 and n-6 polyunsaturated fatty acids. Hepatology. 2015;61(5):1565–1578. 10.1002/hep.27695 25581263

[29] Puri P, Baillie RA, Wiest MM, Mirshahi F, Choudhury J, Cheung O, et al A lipidomic analysis of nonalcoholic fatty liver disease. Hepatology. 2007;46(4):1081–1090. 17654743 10.1002/hep.21763

[30] Depner CM, Traber MG, Bobe G, Kensicki E, Bohren KM, Milne G, et al A Metabolomic Analysis of Omega-3 Fatty Acid-Mediated Attenuation of Western Diet-Induced Nonalcoholic Steatohepatitis in *Ldlr*^*-/-*^ Mice. PLoS One. 2013;8(12):e83756 10.1371/journal.pone.0083756 24358308 PMC3866250

[31] Dixon JB, Bhathal PS, Hughes NR, O'Brien PE. Nonalcoholic fatty liver disease: improvement in liver histological analysis with weight loss. Hepatology. 2004;39(6):1647–1654. 15185306 10.1002/hep.20251

[32] Tilg H, Moschen AR. Evolution of inflammation in nonalcoholic fatty liver disease: the multiple parallel hits hypothesis. Hepatology. 2010;52(5):1836–1846. 10.1002/hep.24001 21038418

[33] Kirschning C, Schumann R. TLR2: cellular sensor for microbial and endogenous molecular patterns Toll-like receptor family members and their ligands: Springer; 2002 p. 121–44. 10.1007/978-3-642-59430-4_8 12467248

[34] Beutler B. TLR4: central component of the sole mammalian LPS sensor. Curr Opin Immuno. 2000;12(1):20–26. 10.1016/s0952-7915(99)00046-1 10679411

[35] Michelsen KS, Doherty TM, Shah PK, Arditi M. TLR signaling: an emerging bridge from innate immunity to atherogenesis. J Immuno. 2004;173(10):5901–5907. 10.4049/jimmunol.173.10.5901 15528321

[36] Taylor KR, Trowbridge JM, Rudisill JA, Termeer CC, Simon JC, Gallo RL. Hyaluronan fragments stimulate endothelial recognition of injury through TLR4. J Biol Chem. 2004;279(17):17079–17084. 14764599 10.1074/jbc.M310859200

[37] Rivera CA, Adegboyega P, van Rooijen N, Tagalicud A, Allman M, Wallace M. Toll-like receptor-4 signaling and Kupffer cells play pivotal roles in the pathogenesis of non-alcoholic steatohepatitis. J Hepato. 2007;47(4):571–579. 10.1016/j.jhep.2007.04.019 PMC2094119 17644211

[38] Moreira APB, Texeira TFS, Ferreira AB, do Carmo Gouveia Peluzio M, de Cássia Gonçalves Alfenas R. Influence of a high-fat diet on gut microbiota, intestinal permeability and metabolic endotoxaemia. Br J Nutr. 2012;108(05):801–809. 22717075 10.1017/S0007114512001213

[39] Harte AL, da Silva NF, Creely SJ, McGee KC, Billyard T, Youssef-Elabd EM, et al Elevated endotoxin levels in non-alcoholic fatty liver disease. J Inflamm. 2010;7:15 10.1186/1476-9255-7-15 PMC2873499 20353583

[40] Liu Y, Han X, Bian Z, Peng Y, You Z, Wang Q, et al Activation of liver X receptors attenuates endotoxin-induced liver injury in mice with nonalcoholic fatty liver disease. Dig Dis Sci. 2012;57(2):390–398. 10.1007/s10620-011-1902-9 21948338

[41] Thuy S, Ladurner R, Volynets V, Wagner S, Strahl S, Königsrainer A, et al Nonalcoholic fatty liver disease in humans is associated with increased plasma endotoxin and plasminogen activator inhibitor 1 concentrations and with fructose intake. J Nutr. 2008;138(8):1452–1455. 18641190 10.1093/jn/138.8.1452

[42] Ghoshal S, Witta J, Zhong J, De Villiers W, Eckhardt E. Chylomicrons promote intestinal absorption of lipopolysaccharides. J Lipid Res. 2009;50(1):90–97. 10.1194/jlr.M800156-JLR200 18815435

[43] Ekstedt M, Hagström H, Nasr P, Fredrikson M, Stål P, Kechagias S, et al Fibrosis stage is the strongest predictor for disease-specific mortality in NAFLD after up to 33 years of follow-up. Hepatology. 2014; 61(5): 1547–1554. 10.1002/hep.27368 25125077

[44] Arthur MJ. Fibrogenesis II. Metalloproteinases and their inhibitors in liver fibrosis. Amer J Physiol-Gastro Liver Physio. 2000;279(2):G245–G249. 10.1152/ajpgi.2000.279.2.G245 10915630

[45] Iredale J, Benyon R, Pickering J, McCullen M, Northrop M, Pawley S, et al Mechanisms of spontaneous resolution of rat liver fibrosis. Hepatic stellate cell apoptosis and reduced hepatic expression of metalloproteinase inhibitors. J Clin Invest. 1998;102(3):538–549. 9691091 10.1172/JCI1018 PMC508915

[46] Perepelyuk M, Terajima M, Wang AY, Georges PC, Janmey PA, Yamauchi M, et al Hepatic stellate cells and portal fibroblasts are the major cellular sources of collagens and lysyl oxidases in normal liver and early after injury. Am J Physiol-Gastrointest Liver Physio. 2013;304(6):G605–G614. 10.1152/ajpgi.00222.2012 PMC3602686 23328207

[47] Ricard-Blum S, Bresson-Hadni S, Guerret S, Grenard P, Volle P, Risteli L, et al Mechanism of collagen network stabilization in human irreversible granulomatous liver fibrosis. Gastroentero. 1996;111(1):172–182. 10.1053/gast.1996.v111.pm8698196 8698196

[48] Mallat A, Lotersztajn S. Cellular mechanisms of tissue fibrosis. 5. Novel insights into liver fibrosis. Am J Physio-Cell Physiol. 2013;305(8):C789–C799. 10.1152/ajpcell.00230.2013 23903700

[49] Moylan CA, Pang H, Dellinger A, Suzuki A, Garrett ME, Guy CD, et al Hepatic gene expression profiles differentiate presymptomatic patients with mild versus severe nonalcoholic fatty liver disease. Hepatology. 2014;59(2):471–482. 10.1002/hep.26661 23913408 PMC3982589

[50] Mattar SG, Velcu LM, Rabinovitz M, Demetris AJ, Krasinskas AM, Barinas-Mitchell E, et al Surgically-induced weight loss significantly improves nonalcoholic fatty liver disease and the metabolic syndrome. Ann Surg. 2005;242(4):618–620. 10.1097/01.sla.0000179652.07502.3f PMC1402345 16192822

[51] Suter M, Calmes J, Paroz A, Giusti V. A 10-year experience with laparoscopic gastric banding for morbid obesity: high long-term complication and failure rates. Obesity Surgery. 2006;16(7):829–835. 16839478 10.1381/096089206777822359

[52] Wouters K, van Gorp PJ, Bieghs V, Gijbels MJ, Duimel H, Lütjohann D, et al Dietary cholesterol, rather than liver steatosis, leads to hepatic inflammation in hyperlipidemic mouse models of nonalcoholic steatohepatitis. Hepatology. 2008;48(2):474–486. 10.1002/hep.22363 18666236

[53] Min H-K, Kapoor A, Fuchs M, Mirshahi F, Zhou H, Maher J, et al Increased hepatic synthesis and dysregulation of cholesterol metabolism is associated with the severity of nonalcoholic fatty liver disease. Cell Met. 2012;15(5):665–674. 10.1016/j.cmet.2012.04.004 PMC3361911 22560219

[54] Janowski BA, Grogan MJ, Jones SA, Wisely GB, Kliewer SA, Corey EJ, et al Structural requirements of ligands for the oxysterol liver X receptors LXRα and LXRβ. Proc Natl Acad Sci. U.S.A. 1999;96(1):266–271. 9874807 10.1073/pnas.96.1.266 PMC15128

[55] Pickens MK, Yan JS, Ng RK, Ogata H, Grenert JP, Beysen C, et al Dietary sucrose is essential to the development of liver injury in the methionine-choline-deficient model of steatohepatitis. J Lipid Res. 2009;50(10):2072–2082. 10.1194/jlr.M900022-JLR200 19295183 PMC2739762

[56] Pagliassotti MJ, Prach PA, Koppenhafer TA, Pan DA. Changes in insulin action, triglycerides, and lipid composition during sucrose feeding in rats. Am J Physiol-Regulatory, Integrat Comp Physiol. 1996;271(5):R1319–R1326. 10.1152/ajpregu.1996.271.5.R1319 8945970

[57] Lim JS, Mietus-Snyder M, Valente A, Schwarz J-M, Lustig RH. The role of fructose in the pathogenesis of NAFLD and the metabolic syndrome. Nature Reviews Gastroenterology and Hepatology. 2010;7(5):251–264. 10.1038/nrgastro.2010.41 20368739

[58] Park HG, Kothapalli KS, Park WJ, DeAllie C, Liu L, Liang A, et al Palmitic acid (16: 0) competes with omega-6 linoleic and omega-3 α-linolenic acids for FADS2 mediated Δ6-desaturation. Biochim Biophys Acta-Molecular and Cell Biology of Lipids. 2015; 186(2): 91–97. 10.1016/j.bbalip.2015.11.007 PMC4691389 26597785

