# Supplementary figures and images for: Is Western Diet-Induced Nonalcoholic Steatohepatitis in Ldlr-/- Mice Reversible?
Source: PLoS One. 2016 Jan 13;11(1):e0146942. doi: 10.1371/journal.pone.0146942 (PMC4711955; doi:10.1371/journal.pone.0146942)

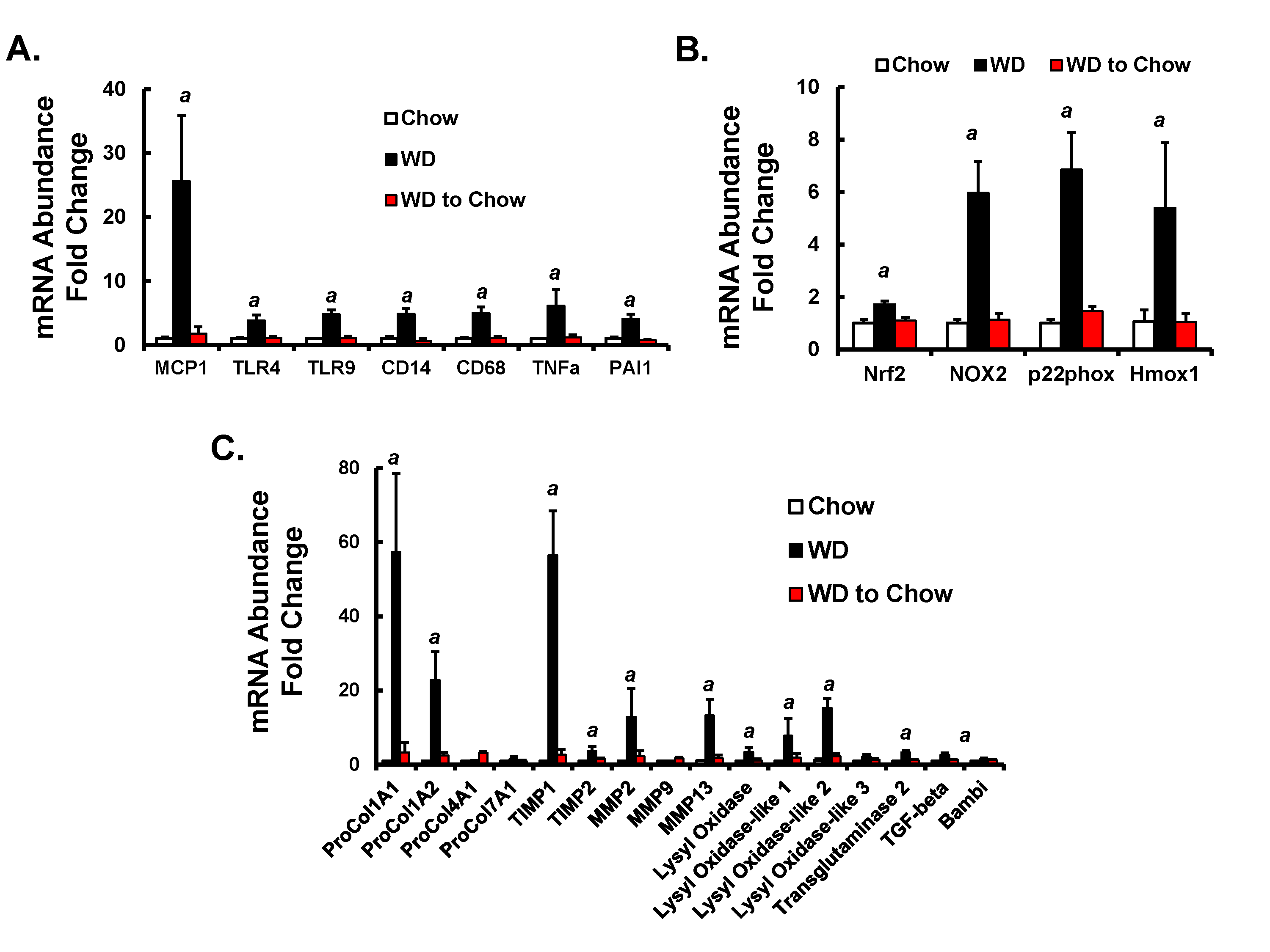

Supplement: S1 Fig — Mice were fed the Chow or WD diets as described in Materials and Methods. RNA was extracted and the abundance of mRNA transcripts encoding proteins involved in inflammation [A], oxidative stress [B] and fibrosis and ECM remodeling [C] were quantified. Results are presented as mRNA Abundance-Fold Change. Results are expressed as mean ± SD with 4 animals/group. a, p≤0.05 versus the chow group. (TIFF) [file pone.0146942.s001.tiff]

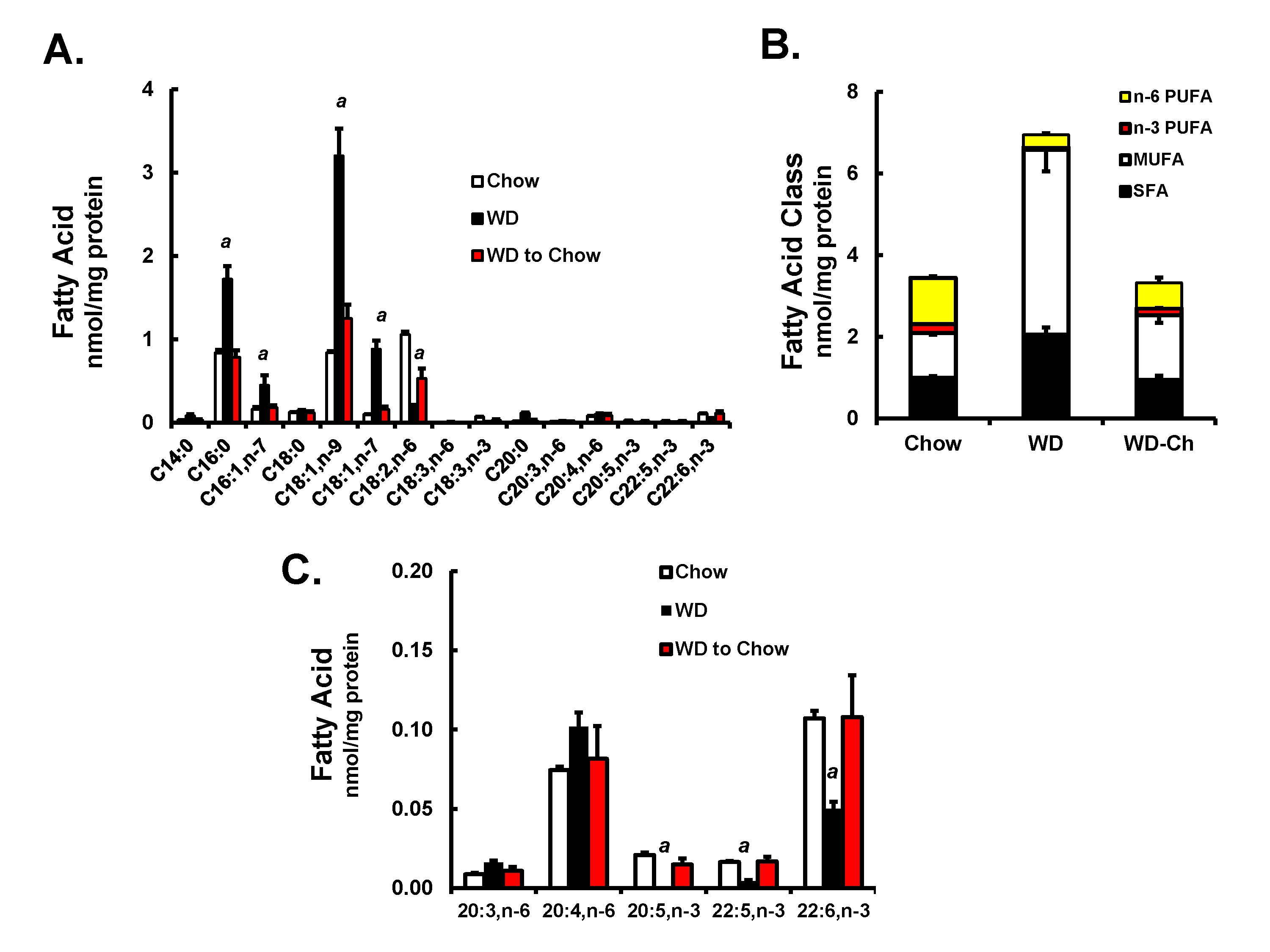

Supplement: S2 Fig — Hepatic lipids were extracted, saponified, converted to fatty acid methyl esters and quantified by gas chromatography as described in Material and Methods. [A]: Hepatic fatty acid content, results are expressed as nmol/mg protein; [B]: Sum of fatty acids in specific classes; results are expressed as nmol/mg protein; [C]: Hepatic C20-22 n-3 and n-6 fatty acids, results are expressed as nmol/mg protein. Results are expressed as mean ± SD with 4 animals/group. a, p≤0.05 versus the chow group. (TIFF) [file pone.0146942.s002.tiff]
