# Supplementary material for: Is Western Diet-Induced Nonalcoholic Steatohepatitis in Ldlr-/- Mice Reversible?
Source: PLoS One. 2016 Jan 13;11(1):e0146942. doi: 10.1371/journal.pone.0146942 (PMC4711955; doi:10.1371/journal.pone.0146942)
Supplement: S1 Table — (DOCX) [file pone.0146942.s003.docx]

**S1 Table Diet Composition**

|  | **Non-Purified (NP) Diet** | **Low Fat-Low Cholesterol (LFLC) Diet** | **Western Diet (WD)** |
| --- | --- | --- | --- |
|  | **Purina** | **Research Diets** | **Research Diets** |
|  | **5350** | **D12450B** | **D12079B** |
| **Composition, Energy%** |  |  |  |
| Protein | 25 | 20 | 17 |
| Carbohydrate | 62 | 70 | 43 |
| Fat | 13 | 10 | 41 |
|  |  |  |  |
| **Energy % as:** |  |  |  |
| Starch + Maltodextrin | 54.2 | 35 | 13.1 |
| Lactose + Glucose | 2.5 |  |  |
| Sucrose + Fructose | 5.5 | 35 | 29.9 |
|  |  |  |  |
| Saturated Fat | 2.6 | 2.7 | 27.4 |
| MUFA | 2.8 | 3.4 | 10.3 |
| ω3 PUFA | 0.9 | 0.4 | 0.3 |
| ω6 PUFA | 6.9 | 3.5 | 1.8 |
| Trans-Fat |  |  | 1.1 |
| Conjugated Linoleic Acid |  |  | 0.16 |
|  |  |  |  |
| **Energy Density, Kcal/g** | 3.4 | 3.9 | 4.7 |
|  |  |  |  |
| **Cholesterol, gm%** | 0.014 | 0.0014 | 0.15 |
|  |  |  |  |
