# Supplementary material for: Is Western Diet-Induced Nonalcoholic Steatohepatitis in Ldlr-/- Mice Reversible?
Source: PLoS One. 2016 Jan 13;11(1):e0146942. doi: 10.1371/journal.pone.0146942 (PMC4711955; doi:10.1371/journal.pone.0146942)
Supplement: S2 Table — (DOCX) [file pone.0146942.s004.docx]

**S2 Table Body weight, plasma and liver parameters-Study1^1^**

| **Feature** | **Units** | **NP-29** | **WD-29** | **WD to NP** |
| --- | --- | --- | --- | --- |
|  |  |  |  |  |
| **Body Weight** | *g* | 28.0 + 1.8^a^ | 44.6 + 2.5^b^ | 31.9 + 3.9^a^ |
|  |  |  |  |  |
| **Plasma Parameters** |  |  |  |  |
| Glucose | *mg/dl* | 114 + 22^a^ | 240 + 62^b^ | 164 + 70^a^ |
| Triglycerides | *mg/dl* | 99 + 36^a^ | 361 + 22^b^ | 142 + 25^a^ |
| Cholesterol | *mg/dl* | 180 + 24^a^ | 1365 + 72^b^ | 279 + 46^a^ |
| ALT | *U/L* | 5.9 + 1.9^a^ | 61.2 + 4.0^b^ | 4.3 + 2.6^a^ |
| AST | *U/L* | 12.9 + 2.6^a^ | 51.8 + 20.3^b^ | 13.2 + 2.0^a^ |
| Leptin | *ng/ml* | 12.8 + 10.5^a^ | 78.9 + 0.7^b^ | 14.4 + 5.8^a^ |
| Adiponectin | *µg/ml* | 6.6 + 0.04^a^ | 5.4 + 0.8^a^ | 6.1 + 1.9^a^ |
| TNFα | *pg/ml* | 0.3 + 0.2^a^ | 8.6 + 2.7^b^ | 0.4 + 0.5^a^ |
| TLR2-Activation | *U/ml* | 20.1 + 3.5^a^ | 59.9 + 10.0^b^ | 23.4 + 2.6^a^ |
| TLR4-Activation | *U/ml* | 33.9 + 8.0^a^ | 82.1 + 8.1^b^ | 44.1 + 11.3^a^ |
| Endotoxin | *EU/ml* | 0.46 + 0.03^a^ | 4.4 + 0.8^b^ | 0.46 + 0.03^a^ |
|  |  |  |  |  |
| **Liver Parameters** |  |  |  |  |
| Liver Weight | *g* | 1.37 + 0.2^a^ | 3.1 + 0.4^b^ | 1.4 + 0.2^a^ |
| Liver Weight | *%BW* | 4.57 + 0.2^a^ | 7.0 + 0.5^b^ | 4.4 + 0.3^b^ |
| Triglyceride | *mg/g protein* | 124.0 + 20.3^a^ | 248.9 + 17.2^b^ | 136.6 + 46.4^a^ |
| Cholesterol | *mg/g protein* | 12.2 + 8.4^a^ | 76.5 + 3.6^b^ | 22.6 + 5.9^a^ |
|  |  |  |  |  |

^1^Values are mean + SD, N=4 mice/treatment group. Labeled means in a row with superscripts

without a common letter differ, *p < 0.05*.
