# Supplementary material for: Is Western Diet-Induced Nonalcoholic Steatohepatitis in Ldlr-/- Mice Reversible?
Source: PLoS One. 2016 Jan 13;11(1):e0146942. doi: 10.1371/journal.pone.0146942 (PMC4711955; doi:10.1371/journal.pone.0146942)
Supplement: S3 Table — (DOCX) [file pone.0146942.s005.docx]

**S3 Table Body weight, plasma and liver parameters-Study 2^1^**

| **Features** | **Units** | **LFLC-32** | **WD-24** | **WD-24 to LFLC-8** | **WD-32** |
| --- | --- | --- | --- | --- | --- |
|  |  |  |  |  |  |
| **Body Weight** | *g* | 34.6 + 4.8^a^ | 39.3 + 2.4^b^ | 34.8 + 3.5^a^ | 42.1 + 4.9^c^ |
|  |  |  |  |  |  |
| **Plasma Parameters** |  |  |  |  |  |
| Glucose | *mg/dl* | 150 + 33^a^ | 293 + 51^b^ | 165 + 32^a^ | 302 + 31^b^ |
| Triglycerides | *mg/dl* | 135 + 48^a^ | 327 + 64^b^ | 167 + 49^a^ | 381 + 54^b^ |
| Cholesterol | *mg/dl* | 515 + 109^a^ | 1286 + 289^b^ | 529 + 219^a^ | 1674 + 510^b^ |
| ALT | *U/L* | 7.9 + 2.7^a^ | 30.4 + 13.8^b^ | 10.9 + 6.4^a^ | 28.5 + 2.7^b^ |
| Leptin | *ng/ml* | 24.7 + 10.5^a^ | 44.4 + 9.0^b^ | 25.3 + 7.7^a^ | 63.9 + 8.8^c^ |
| Adiponectin | *µg/ml* | 7.3 + 0.3^a^ | 5.5 + 2.3^a^ | 6.9 + 0.4^a^ | 5.0 + 2.1^a^ |
| TLR2-Activation | *U/ml* | 17 + 5.4^a^ | 38.9 + 8.1^b^ | 17.6 + 2.7^a^ | 35.2 + 13.8^b^ |
| TLR4-Activation | *U/ml* | 35.5 + 7.5^a^ | 56 + 10.1^b^ | 35.1 + 4.1^a^ | 50.0 + 11.3^b^ |
|  |  |  |  |  |  |
| **Liver Parameters** |  |  |  |  |  |
| Liver Weight | *g* | 1.3 + 0.2^a^ | 2.3 + 0.4^b^ | 1.7 + 0.4^a^ | 2.7 + 0.8^b^ |
| Liver Weight | *%BW* | 4.1 + 0.3^a^ | 5.7 + 0.9^b^ | 4.9 + 0.6^b^ | 6.3 + 1.2^c^ |
| Triglyceride | *mg/g protein* | 174.0 + 40.8^a^ | 275.3 + 41.1^b^ | 329.4 + 72.3^b^ | 301.6 + 57.9^b^ |
| Cholesterol | *mg/g protein* | 21.0 + 3.5^a^ | 44.5 + 5.6^b^ | 48.3 + 12.9^b^ | 50.5 + 16.1^b^ |

^1^Values are mean + SD, N-5-6/treatment group. Labeled means in a row with superscripts without a common letter differ, *p < 0.05*.
