# Supplementary material for: Is Western Diet-Induced Nonalcoholic Steatohepatitis in Ldlr-/- Mice Reversible?
Source: PLoS One. 2016 Jan 13;11(1):e0146942. doi: 10.1371/journal.pone.0146942 (PMC4711955; doi:10.1371/journal.pone.0146942)
Supplement: S4 Table — (DOCX) [file pone.0146942.s006.docx]

**S4 Table Comparison of NP- versus LFLC-fed *Ldlr^-/-^* mice.^1^**

| **Features** | **Units** | **NP-29** | **LFLC-32** |
| --- | --- | --- | --- |
|  |  |  |  |
| **Body Weight** | *g* | 28.0 + 1.8^a^ | 34.6 + 4.8^a^ |
|  |  |  |  |
| **Plasma Parameters** |  |  |  |
| Glucose | *mg/dl* | 114 *+* 22^a^ | 150 + 33^a^ |
| Triglycerides | *mg/dl* | 99 + 36^a^ | 135 + 48a |
| Cholesterol | *mg/dl* | 180 + 24^a^ | 515 + 109^b^ |
| ALT | *U/L* | 5.9 + 1.9^a^ | 7.9 + 2.7^a^ |
| Leptin | *ng/ml* | 12.8 + 10.5^a^ | 24.7 + 10.5^a^ |
| Adiponectin | *µg/ml* | 6.6 + 0.04^a^ | 7.3 + 0.3^a^ |
| TLR2-Activation | *U/ml* | 20.1 + 3.5^a^ | 17 + 5.4^a^ |
| TLR4-Activation | *U/ml* | 33.9 + 8.0^a^ | 35.5 + 7.5^a^ |
|  |  |  |  |
| **Liver Parameters** |  |  |  |
| Liver Weight | *g* | 1.37 + 0.07^a^ | 1.3 + 0.2a |
| Liver Weight | *%BW* | 4.57 + 0.06^a^ | 4.1 + 0.3^a^ |
| Triglyceride | *mg/g protein* | 124.0 + 20.3^a^ | 174.0 + 40.8^a^ |
| Cholesterol | *mg/g protein* | 12.2 + 8.4^a^ | 21.0 + 3.5^a^ |
|  |  |  |  |
| **Liver Gene Expression** |  |  |  |
| *Col1A1* |  | 1.0 + 0.09^a^ | 7.87 + 7.6^b^ |
| *Timp1* |  | 1.0 + 0.06^a^ | 5.23 + 3.2^b^ |
| *Mcp1* |  | 1.0 + 0.25^a^ | 8.53 + 6.1^b^ |
| *Nox2* |  | 1.0 + 0.13^a^ | 2.0 + 0.9^a^ |
|  |  |  |  |

^1^Values are mean + SD, N= >4 mice/treatment group. Labeled means in a row with

superscripts without a common letter differ, *p < 0.05*.
