# Supplementary material for: Is Western Diet-Induced Nonalcoholic Steatohepatitis in Ldlr-/- Mice Reversible?
Source: PLoS One. 2016 Jan 13;11(1):e0146942. doi: 10.1371/journal.pone.0146942 (PMC4711955; doi:10.1371/journal.pone.0146942)
Supplement: S5 Table — (DOCX) [file pone.0146942.s007.docx]

**S5 Table Significant features associated with NASH in *Ldlr^-/-^* mice as determined by ANOVA-Tukey HSD^1^.**

| **Feature** | ***p-value*** |  | **Feature** | ***p-value*** |
| --- | --- | --- | --- | --- |
| Plasma TLR2 Agonist | 3.7209E-10 |  | *TLR9* | 2.5867E-05 |
| *Loxl2* | 6.0637E-10 |  | 18:1,ω9 | 3.5336E-05 |
| Plasma Triglyceride | 1.7094E-09 |  | Hepatic Total Cholesterol | 4.5044E-05 |
| *NOX2* | 7.9546E-09 |  | Plasma TLR4 Agonist | 5.6484E-05 |
| *TIMP1* | 2.3379E-08 |  | Plasma AST | 0.00010591 |
| Plasma Free Cholesterol | 2.5266E-08 |  | Hepatic SFA | 0.00016325 |
| 20:0 | 1.2056E-07 |  | Palmitate | 0.00017799 |
| 18:2,ω6 | 2.0071E-07 |  | Body Weight | 0.00017813 |
| *Hmox1* | 0.000000233 |  | Sum Fatty Acids | 0.00022453 |
| 18:3,ω6 | 2.4676E-07 |  | 16:1,ω7 | 0.00025218 |
| *Mcp1* | 2.4765E-07 |  | *TGH* | 0.00028617 |
| *TGFα* | 8.8663E-07 |  | Plasma Leptin | 0.00054225 |
| ω6 PUFA | 1.0036E-06 |  | 20:3,ω6 | 0.00070021 |
| *TIMP2* | 1.2621E-06 |  | *ProCol1A1* | 0.00094686 |
| Plasma Total Cholesterol | 1.4716E-06 |  | 20:4,ω6 | 0.0012236 |
| ω3 PUFA | 2.0693E-06 |  | *Scd1* | 0.0012716 |
| Plasma TLR4 agonist | 2.0741E-06 |  | Hepatic Free Cholesterol | 0.0015634 |
| *Elovl5* | 2.1354E-06 |  | Adiponectin | 0.0018532 |
| 18:1,ω7 | 2.2255E-06 |  | PPARλ | 0.0030989 |
| *Lox* | 2.5978E-06 |  | Stearate | 0.0031297 |
| *Elovl7* | 4.4971E-06 |  | *ATGL* | 0.0033301 |
| Liver weight | 5.1041E-06 |  | *Mmp13* | 0.0038285 |
| *CD68* | 0.000005158 |  | *Fads1* | 0.0076763 |
| Liver Weight % Body Weight | 6.5556E-06 |  | *Mmp2* | 0.0079912 |
| 18:3,ω3 | 7.6474E-06 |  | *Clec4f* | 0.010175 |
| 22:6,ω3 | 0.000012979 |  | Hepatic Triglyceride | 0.01266 |
| Plasma ALT | 0.0000164 |  | Plasma β-hydroxybutyrate | 0.01513 |
| Hepatic MUFA | 0.000022329 |  | *LoxL1* | 0.031686 |

^1^All data used to construct the heat map in **Fig. 5** was used for statistical analysis using the MetaboAnalyst 3.0 statistical package. The table above lists the all features with *p-values < 0.05*. Significant features associated with NASH in *Ldlr^-/-^* mice were determined by ANOVA-Tukey HSD”.
